# Harnessing Controlled Dealloying–Support Coupling for Ultrastable PtNi Catalysts in PEMFC Applications

**DOI:** 10.1002/anie.4524344

**Published:** 2026-02-09

**Authors:** Fei Guo, Manxi Gong, Longxiang Liu, Bochen Li, Ruwei Chen, Mengjun Gong, Wei Zong, Jianuo Chen, Qi Li, Jing Li, Yunpeng Zhong, Zeyi Zhang, Jianrui Feng, Rhodri Jervis, Guanjie He

**Affiliations:** ^1^ Department of Chemistry University College London London UK; ^2^ Department of Materials University of Oxford Oxford UK; ^3^ Electrochemical Innovation Lab Department of Chemical Engineering University College London London UK; ^4^ Department of Chemistry Imperial College London White City Campus London UK; ^5^ Department of Chemistry School of Physical and Chemical Sciences Queen Mary University of London London UK; ^6^ Department of Chemistry University of Zurich Zurich Switzerland

## Abstract

Platinum–transition metal (PtM) alloys are among the most promising oxygen reduction reaction (ORR) catalysts, yet their practical deployment in proton‐exchange membrane fuel cells (PEMFCs) is hindered by transition‐metal dissolution, particle coarsening, and insufficient durability. Moreover, conventional alloying or intermetallic ordering strategies often aggravate these issues by inducing severe nanoparticle aggregation and instability. Here we report a controllable alloying–dealloying strategy to construct PtNi nanoparticles confined in an N‐doped carbon framework (Pt_1_Ni_1‐x_@Ni_x__NC). Ammonia‐assisted dealloying produces a Pt‐rich shell with an alloyed core, while the N‐doped carbon anchors the released Ni atoms form Ni–N/C moieties, thereby suppressing agglomeration and strengthening metal–support interactions. This coordination–support coupling optimizes Pt 5d orbital occupation, weakens oxygen adsorption, and accelerates ORR kinetics. Consequently, Pt_1_Ni_1‐x_@Ni_x__NC exhibits a half‐wave potential of 0.932 V and an ultrahigh mass activity of 2.028 A mgPt^−1^, which is 8.75‐fold higher than commercial Pt/C and among the best values reported to date for PtNi‐based catalysts. Remarkably, it shows only a 6 mV half‐wave potential loss after 30,000 cycles, demonstrating exceptional durability. In PEMFCs, the fuel cell delivers 975 mW cm^−2^ peak power density and retains 91.9% of initial performance, underscoring a generalizable approach for designing durable, high‐performance low‐PGM catalysts for next generation PEMFCs.

## Graphical abstract

A controllable alloying–dealloying strategy constructs PtNi core–shell nanoparticles confined within an N‐doped carbon framework. Selective ammonia‐assisted dealloying forms a Pt‐rich shell while immobilizing Ni as Ni–N/C moieties, synergistically stabilizing the catalyst. The resulting structure delivers exceptional ORR activity and durable, high‐power performance in practical PEM fuel cells.

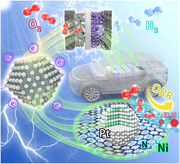

## 1 Introduction

Proton‐exchange membrane fuel cells (PEMFCs) have emerged as promising energy‐conversion devices owing to their low operating temperature, high efficiency, and environmentally benign operation [1, 2, 3]. These advantages position PEMFCs as a critical power source for commercial transportation, offering a sustainable alternative to fossil fuels and accelerating progress toward a carbon‐neutral economy [4, 5]. Nevertheless, practical deployment requires electrocatalysts that simultaneously deliver high power density and long‐term durability. The performance and lifetime of PEMFCs are largely dictated by the cathode catalyst, where the inherently sluggish oxygen reduction reaction (ORR) remains the principal bottleneck [6, 7, 8]. In addition, water generated during the ORR tends to accumulate at the cathode under low‐temperature operating conditions, causing electrode flooding if not effectively regulated. Accordingly, the design of cathode electrocatalysts with high intrinsic activity, robust structural stability, and efficient water management capabilities is essential for enabling the widespread implementation of PEMFCs technologies [9, 10].

To date, most state‐of‐the‐art ORR electrocatalysts are based on platinum (Pt) nanoparticles supported on carbon materials, with commercial Pt/C serving as the benchmark. However, the intrinsic limitations of Pt/C, insufficient catalytic activity, and poor durability continue to hinder large‐scale fuel‐cell deployment [11]. Alloying Pt with 3d transition metals (M = Co [12, 13], Ni [14, 15], Fe [16, 17]) has emerged as an effective strategy to enhance ORR performance while reducing Pt usage. The incorporation of these transition metals modulates the Pt 5d‐band center, optimizes the adsorption energy of oxygenated intermediates, and accelerates ORR kinetics. Yet, under the harsh operating conditions of PEMFCs, these transition metals are prone to dissolution, resulting in progressive degradation of catalytic activity and stability. Moreover, the leached metal ions further impede proton transport within the membrane [18, 19]. To mitigate these drawbacks, ordered PtM intermetallic compounds [20] core‐shell Pt@PtM nanostructures [21, 22, 23, 24] have been developed, offering improved structural stability and tunable surface electronic properties. Despite their promise, the synthesis of ordered PtM intermetallics remains challenging due to Ostwald ripening during high‐temperature annealing, which drives nanoparticle migration and coalescence, ultimately enlarging particle size [25, 26]. Such particle growth drastically reduces the electrochemically active surface area and compromises catalytic performance. Therefore, engineering strong interactions between the nanoparticle catalyst and its support is imperative to suppress over‐aggregation during thermal treatment, thereby enabling the formation of highly dispersed, stable, and catalytically active PtM nanostructures [27]. Furthermore, the structural confinement of PtM nanoparticles effectively suppresses nanoparticle migration and agglomeration, ensuring the long‐term stability of active sites during prolonged electrochemical cycling in PEMFCs. Recent studies have shown that deliberately constructing interfacial coordination motifs on carbon supports—such as B–N–C moieties in turbostratic carbon or ligand‐coordinated metal–carbon interfaces—can effectively immobilize metal species and suppress migration‐induced degradation by blocking metal diffusion pathways and stabilizing local coordination environments [28, 29].

Herein, we report a controllable alloying–dealloying strategy to construct PtNi nanoparticles confined within an N‐doped carbon matrix (denoted as Pt_1_Ni_1‐x_@Ni_x__NC). Guided by theoretical predictions, selective ammonia‐assisted dealloying transforms disordered PtNi alloys into core–shell nanoparticles featuring a Pt‐rich shell and PtNi alloyed core, while simultaneously anchoring dissolved Ni atoms onto the N‐doped carbon framework to form Ni_NC moieties. This dual stabilization not only preserves particle dispersion during high‐temperature treatment but also optimizes the surface electronic configuration of Pt, thereby accelerating ORR kinetics. Structural and spectroscopic characterizations reveal that this coordinated evolution modulates the Pt 5d orbital occupation, weakens oxygen adsorption, and enhances interfacial water management Benefiting from the synergistic alloy–support coupling, Pt_1_Ni_1‐x_@Ni_x__NC achieves a half‐wave giotential of 0.932 V and a record‐high mass activity of 2.028 A mgPt^−1^—8.75 times higher than commercial Pt/C and exceeding most state‐of‐the‐art Pt‐based catalysts reported to date. Moreover, in membrane electrode assemblies (MEAs), the catalyst delivers a peak power density of 975 mW cm^−2^ and retains 91.9% of its initial performance after 30,000 cycles, demonstrating exceptional operational robustness under realistic PEMFCs conditions. This work establishes a generalizable pathway that integrates controllable alloying–dealloying chemistry with heteroatom‐doped carbon confinement to produce low‐PGM catalysts combining unprecedented activity and durability for next‐generation fuel‐cell technologies.

## 2 Result and Discussion

To accurately evaluate the catalytic performance of nanoparticles with distinct structural configurations in the four‐electron oxygen reduction reaction (ORR), three representative models were constructed: pure Pt, a PtNi intermetallic compound (noted as Pt_3_Ni), and a PtNi intermetallic core encapsulated by a Pt shell (noted as Pt@Pt_3_Ni). Density functional theory (DFT) calculations were employed to investigate their electronic structures and to assess the adsorption and desorption characteristics of key ORR intermediates (*OOH, *O, and *OH) along the complete four‐electron reaction pathway. This theoretical framework enables a deeper understanding of the structure–property relationships governing the ORR activity and provides guidance for the rational design of high‐performance electrocatalysts.

**FIGURE 1:**
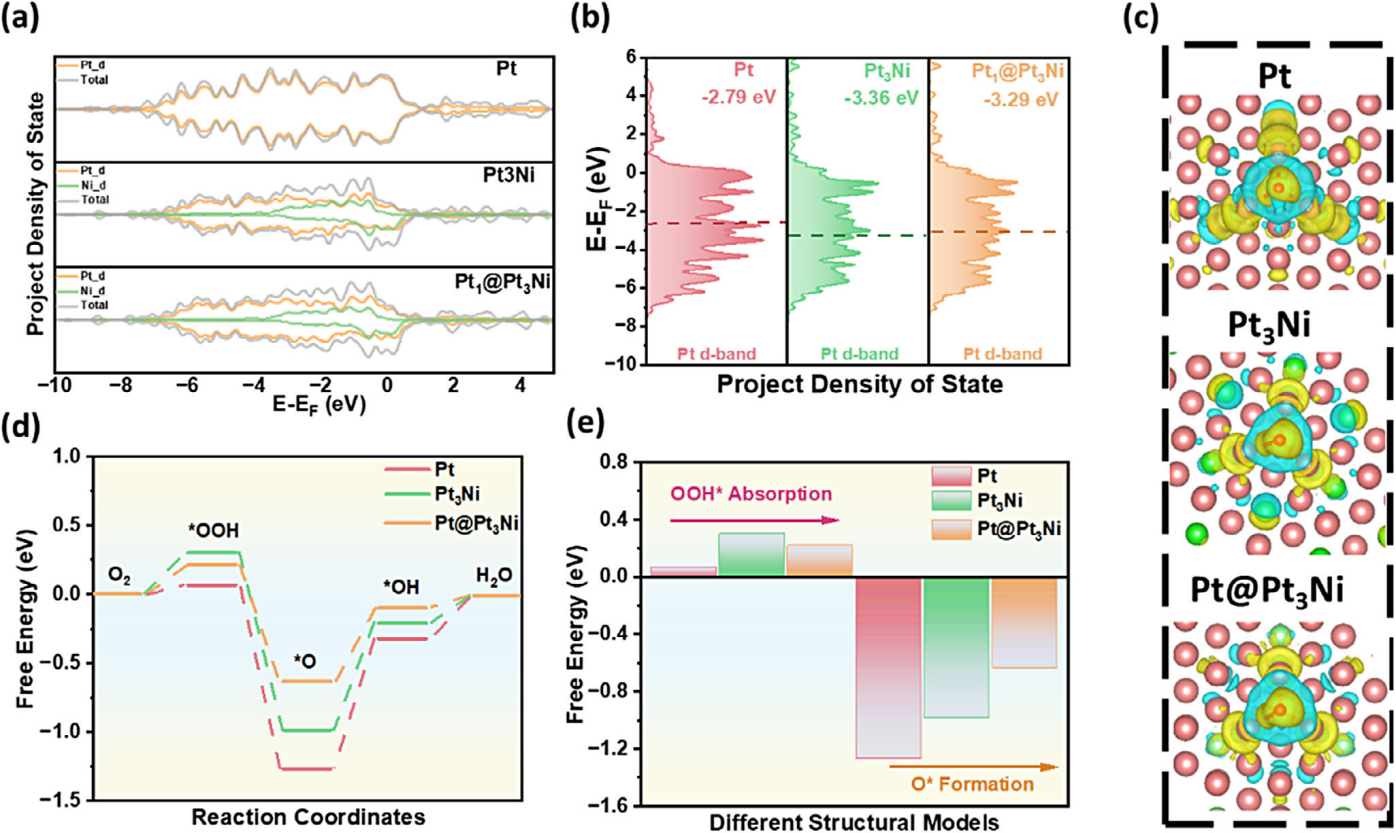
(a) The total and corresponding d states of Pt, Pt_3_Ni, and Pt@Pt_3_Ni. (b) d‐Band center of Pt, Pt_3_Ni, and Pt@Pt_3_Ni. (c) The electron density difference of Pt, Pt_3_Ni, and Pt@Pt_3_Ni, where blue and yellow represent areas of electron diminishing and accumulation; red atoms and green atoms represent platinum and nickel, respectively. (d) Calculated 4e^−^ reaction pathway of ORR on Pt, Pt_3_Ni, and Pt@Pt_3_Ni. (e) Corresponding free energy of OOH* absorption and O* formation on different models.

The d‐band center serves as a crucial link between the electronic structure of a catalyst and its catalytic performance. To investigate how structural variations in different nanoparticle models influence the d‐band characteristics, projected density of states (PDOS) calculations were conducted. These analyses also provide insight into the contributions of individual elements to the total electronic states. As shown in Figure 1a, both Pt_3_Ni and Pt@Pt_3_Ni exhibit newly formed hybridized electronic states compared to pure Pt. These states likely arise from the interaction between the valence electrons of Pt atoms and the neighboring Ni atoms. Moreover, the overall density of states is noticeably modulated by the nanostructure, with a continuous peak near the Fermi level (E_F_), suggesting enhanced electrochemical adsorption and desorption capabilities. A notable observation is that in all models, the density of states in the energy region below the E_F_ (i.e., the occupied states) exhibits higher intensity and greater enrichment compared to the unoccupied states above E_F_. This indicates that Pt‐based nanostructures can supply ample electrons to facilitate the ORR, thus supporting rapid reaction kinetics [30, 31]. In particular, Pt_3_Ni and Pt@Pt_3_Ni demonstrate significant shifts in their PDOS profiles relative to pure Pt, attributed to strong d‐orbital hybridization between Pt and Ni atoms. As illustrated in Figure 1b, this electronic coupling results in a shift of the Pt d‐band center (E_d‐band_). For pure Pt, E_d is located at −2.79 eV, while for Pt_3_Ni, it shifts to −3.36 eV, indicating that more electrons occupy anti‐bonding states. Although this enhances the absorption of reaction intermediates, it consequently hinders the desorption. Interestingly, the E_d‐band_ of Pt@Pt_3_Ni lies at −3.29 eV, a position that suggests a more balanced electronic environment. This intermediate shift allows for simultaneous optimization of both adsorption and desorption steps in the ORR pathway, thereby promoting favorable reaction kinetics and improved catalytic efficiency. The charge density difference results (Figure 1c) reveal the presence of extensive electron transfer channels between Pt and Ni atoms. Notably, in the Pt@Pt_3_Ni structure, a significant accumulation of electrons is observed around the outer‐layer Pt atoms. This indicates that structural modulation at the nanoscale enables optimization of the surface electronic configuration of Pt through strong electronic interactions with the underlying Ni, thereby enhancing its catalytic potential.

To evaluate the enhancement in ORR kinetics resulting from structural optimization, free energy diagrams for the four‐electron ORR pathway were calculated for Pt, Pt_3_Ni, and Pt@Pt_3_Ni (Figure 1d). For pure Pt, the formation of the *OOH intermediate in the initial step proceeds with relatively low free energy (0.068 eV), indicating a thermodynamically favorable process. However, the subsequent adsorption of *O requires significantly higher energy (−1.268 eV in Figure 1e and Table S1), identifying this step as the rate‐determining step (RDS) that limits the overall catalytic efficiency of pure Pt. In contrast, both Pt_3_Ni and Pt@Pt_3_Ni exhibit stronger *OOH adsorption compared to pure Pt, which suggests more difficult desorption of this intermediate. Nonetheless, they demonstrate substantially lower free energy requirements for the adsorption of *O (−0.986 and −0.634 eV for Pt_3_Ni and Pt@Pt_3_Ni, respectively), indicating a reduced energy barrier for the RDS. Notably, Pt@Pt_3_Ni displays the most favorable overall energy profile along the ORR pathway, with the smallest free energy change among the models studied. These results underscore the critical influence of nanostructure engineering on ORR performances. The unique core–shell architecture of Pt@Pt_3_Ni, comprising a Pt shell and a Pt_3_Ni intermetallic core, modulates the Pt d‐orbital electronic structure via strong Pt–Ni electronic coupling. This facilitates electron transfer to the surface Pt active sites, enabling a more balanced adsorption and desorption of ORR intermediates and ultimately promoting more efficient reaction kinetics.

**FIGURE 2:**
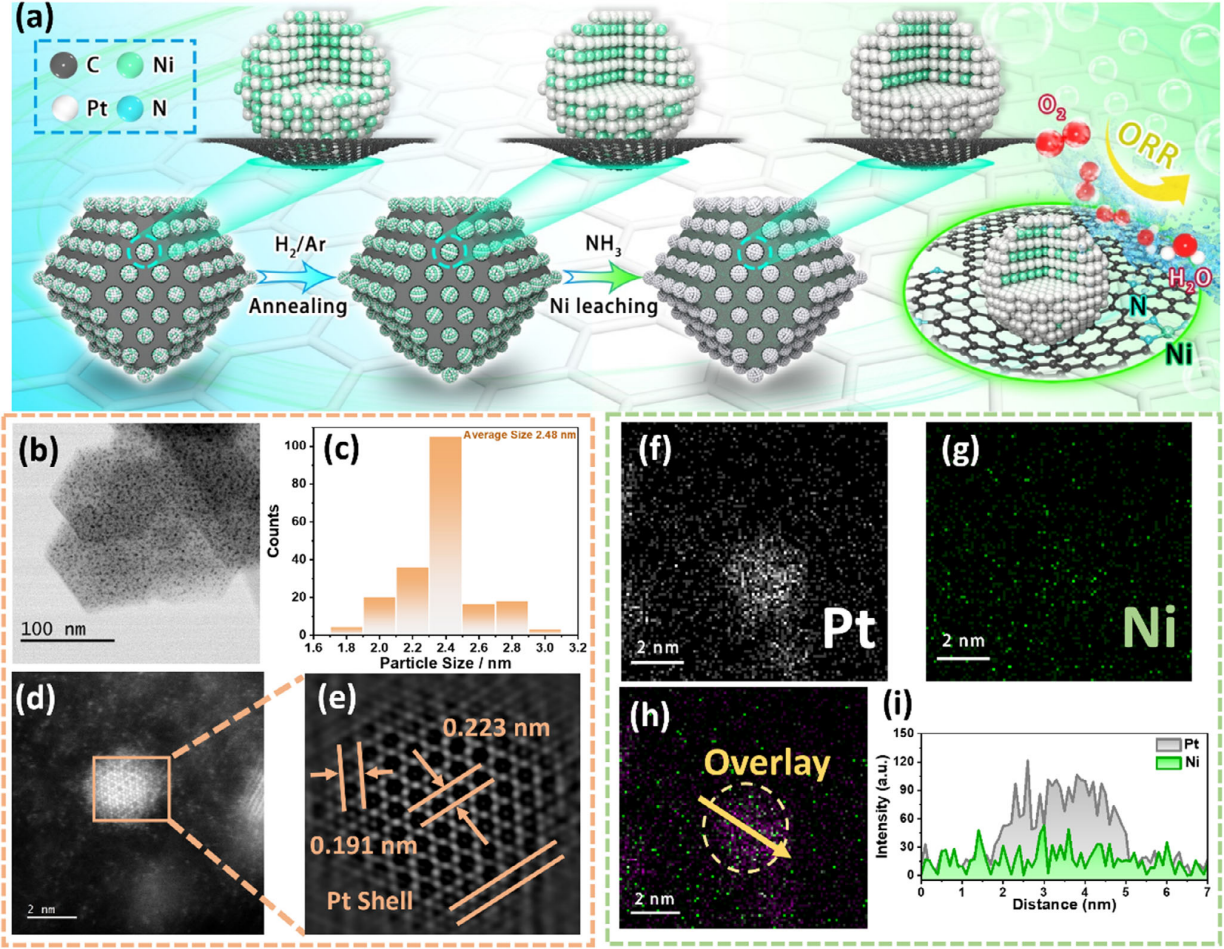
(a) Schematic illustrations of synthesising Pt_1_Ni_1_@NC and Pt_1_Ni_1‐n_@Ni_n__NC. (b) BF‐TEM image of Pt_1_Ni_1‐x_@Ni_x__NC. (c) Size distribution of the nanoparticles from (b). (d) HADDF‐STEM image of Pt_1_Ni_1‐x_@Ni_x__NC. (e) Inverse FFT image from (d). (f, g) EDS mapping of Pt and Ni from the nanoparticle of Figure S6b. (h) Overlap mapping of nanoparticles in Figure S6b. (i) Line scan single collected from the yellow arrow of (h).

To realize the controllable nanoparticle structures (Figure 1) and enhance the overall electrochemical performances of the catalyst, nitrogen‐doped carbon (NC) was employed as the support. NC was derived from the pyrolysis of ZIF‐8, uniformly distributed nanostructures with an average diameter of approximately 70 nm were obtained (Figure S1a,b). As shown in the x‐ray diffraction (XRD) pattern (Figure S1c), no characteristic diffraction peak corresponding to metallic phases is observed, indicating the absence of crystalline metal particles in NC. This observation is further supported by transmission electron microscopy (TEM) analysis (Figure S1d,e), where both bright‐field (BF) and high‐angle annular dark‐field (HAADF) images reveal no discernible metal nanoparticles. The structural model, illustrated in Figure S1f, suggests that the material consists of a nanocarbon cage featuring a highly porous architecture. As illustrated in Figure 2a, Pt^4^^+^ and Ni^2^^+^ cations were introduced into the NC support via an impregnation method, followed by thermal treatment under a H_2_/Ar atmosphere at 750°C to form PtNi alloy nanoparticles, denoted as Pt_1_Ni_1_@NC. Subsequently, the Pt_1_Ni_1_@NC underwent a second heat treatment under flowing NH_3_ at 100°C to induce partial dealloying, designated as Pt_1_Ni_1‐x_@Ni_x__NC. Scanning electron microscopy (SEM) images (Figure S2) reveal that the morphology and particle size of NC support remain largely unchanged after the multi‐step thermal treatment, indicating its excellent thermal stability as a support material. Moreover, the absence of discernible nanoparticles on the NC surface suggests that the porous architecture and nitrogen‐rich composition of NC effectively suppress the aggregation of PtNi particles during both the alloying and partial dealloying processes. To further investigate the structural evolution, TEM was employed to characterize the Pt_1_Ni_1_ and Pt_1_Ni_1‐x_ nanoparticles obtained at various synthesis stages. BF‐TEM image (Figure S3a) confirms the successful synthesis of uniformly distributed PtNi alloy nanoparticles, with an average diameter of 2.54 nm (Figure S3b), confined within the porous structure of the NC support. High‐resolution scanning transmission electron microscopy (HRSTEM) image (Figure S3c), along with the corresponding fast Fourier transform (FFT) patterns (Figure S3d), reveal well‐defined atomic arrangements and distinct diffraction spots, indicative of high crystallinity in the PtNi nanoalloys. Further analysis using HAADF imaging (Figure S3e) and inverse FFT (Figure S3f) shows dominant lattice spacings of 0.213 and 0.186 nm, corresponding to the (111) and (200) planes of face‐centered cubic (fcc) Pt, respectively. The observed slight lattice contraction is attributed to compressive strain induced by the incorporation of smaller Ni atoms into the Pt lattice during the alloy formation. As shown in the BF‐TEM image (Figure 2b), ammonia annealing does not alter the dispersion of the alloy clusters, and the particle size remains uniform, with an average diameter of 2.43 nm (Figure 2c). The HAADF‐STEM image (Figure 2d) reveals the presence of surface defects on the nanoparticles, attributed to the selective dissolution and migration of Ni atoms from the PtNi surface under the ammonia atmosphere. Despite this surface restructuring, the inverse FFT image (Figure 2e) indicates that the majority of the nanoparticle lattice remains well ordered, consistent with Figure S4a, while a distinct Pt‐rich shell forms at the outer edge. Notably, the measured lattice spacings of Pt_1_Ni_1‐x_ nanoparticles (0.223 nm and 0.191 nm) are slightly larger than those of Pt_1_Ni, consistent with the release of compressive lattice strain caused by surface Ni leaching. XRD analysis (Figure S4b) further confirms that the bulk crystal structures of Pt_1_Ni_1‐x_ and Pt_1_Ni_1_ remain largely unchanged following dealloying, with no significant shift in peak positions. These results collectively demonstrate that the dealloying process is effectively confined to the nanoparticle surface under the applied synthesis conditions. In addition, it is noteworthy that the Ni atoms dissolved and migrated during ammonia‐induced dealloying are not lost from the material but are instead recaptured by the NC support. As highlighted by the red circle in Figure S5a, Ni is uniformly distributed in the vicinity of the Pt_1_Ni_1‐x_ nanoparticles. The corresponding BF‐STEM image (Figure S5b) shows no evidence of aggregated Ni nanoparticles or clusters, indicating that the recaptured Ni is dispersed atomically on the NC surface. This observation is further corroborated by elemental line‐scan analysis (Figure S6), which confirms the spatial distribution of Pt and Ni within and around the nanoparticles. As shown in Figure 2f,g, Pt is predominantly enriched at the nanoparticle surface, while Ni exhibits a more diffuse distribution. The overlapped EDS mapping in Figure 2h reveals that the purple regions (overlapping Pt and Ni signals) correspond to the PtNi alloy core, while the green spots surrounding the particles represent Ni atoms redistributed onto the NC support during dealloying. As illustrated in Figure 2i, the linear EDS profile along the yellow arrow (Figure 2h) further confirms a core–shell architecture, with a PtNi alloy core, a Pt‐enriched shell, and Ni species dispersed uniformly around the nanoparticles on the carbon matrix.

**FIGURE 3:**
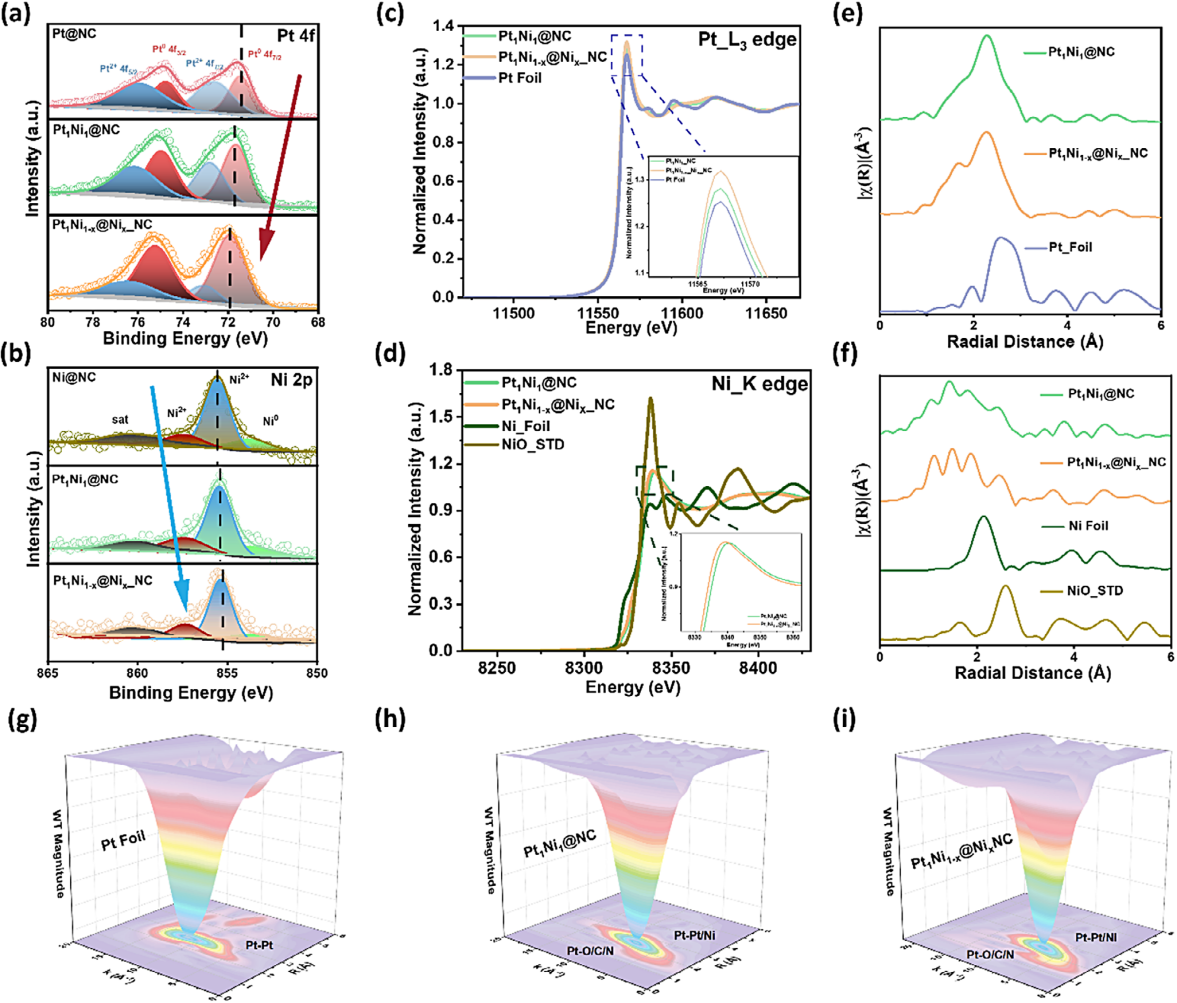
XPS spectra of (a) Pt 4f of Pt@NC, Pt_1_Ni_1_@NC and Pt_1_Ni_1‐x_@Ni_x__NC. (b) Ni 2p of Ni@NC, Pt_1_Ni_1_@NC and Pt_1_Ni_1‐x_@Ni_x__NC. XANES spectra of (c) Pt_L_3_ edge XANES of Pt Foil, Pt_1_Ni_1_@NC and Pt_1_Ni_1‐x_@Ni_x__NC (inset: enlarged absorption near edge peak of Pt_L_3_). (d) Ni_K edge XANES of Ni foil, NiO, Pt_1_Ni_1_@NC, and Pt_1_Ni_1‐x_@Ni_x__NC (inset: enlarged absorption near edge peak of Ni_K edge for Pt_1_Ni_1_@NC and Pt_1_Ni_1‐x_@Ni_x__NC). (e) the k^2^‐weighted 𝛸(k)‐function of the Pt L_3_‐edge EXAFS spectra from (c). (f) The k^2^‐weighted 𝛸(k)‐function of the Ni K edge EXAFS spectra from (d). (g–i) WT‐EXAFS contour maps of the Pt L_3_‐edge for Pt foil, Pt_1_Ni_1_@NC, and Pt_1_Ni_1‐x_@Ni_x__NC.

X‐ray photoelectron spectroscopy (XPS) was employed to investigate the surface electronic structure evolution associated with structural changes in the PtNi alloy catalyst. The high‐resolution Pt 4f spectrum (Figure 3a) reveals that Pt_1_Ni_1‐x_@Ni_x__NC exhibits the highest binding energies among the three samples—Pt@NC, Pt_1_Ni_1_@NC, and Pt_1_Ni_1‐x_@Ni_x__NC. Two distinct Pt 4f doublets are observed at 72.10/75.42 eV and 73.26/76.58 eV (Table S2), corresponding to the Pt^0^ and Pt^2^^+^ oxidation states, respectively. In comparison, Pt@NC and Pt_1_Ni_1_@NC show Pt 4f peaks shifted toward lower binding energies. This positive shift in Pt_1_Ni_1‐x_@Ni_x__NC is attributed to electronic interactions with Ni and structural changes associated with the formation of a core–shell configuration, which leads to an increased Pt 4f binding energy. Moreover, the proportion of metallic Pt^0^ is significantly enhanced in Pt_1_Ni_1‐x_@Ni_x__NC, with a Pt^0^/Pt^2+^ ratio of 2.63, considerably higher than those of Pt@NC (0.71) and Pt_1_Ni_1_@NC (1.26) (Figure S7), indicating a more electron‐rich Pt surface environment. This modulation of the surface electronic structure is further supported by the Ni 2p XPS spectra (Figure 3b, Table S3). In Pt_1_Ni_1‐x_@Ni_x__NC, the Ni 2p peaks exhibit a negative shift compared to Ni@NC, consistent with the trend observed in the Pt 4f spectra. This shift confirms strong electronic interactions between Pt and Ni, which are further enhanced after dealloying and core–shell structure formation. Notably, three characteristic Ni 2p features are observed in Figure 3b, corresponding to Ni^0^, Ni^2+^ (from partially oxidized Ni species), and another Ni^2+^ species likely associated with nitrogen coordination in the NC support, indicating the coexistence of multiple chemical states of Ni in the system.

X‐ray absorption near‐edge spectroscopy (XANES) and extended x‐ray absorption fine structure (EXAFS) measurements were carried out to probe the electronic structure and local coordination environment of PtNi nanoparticles before and after dealloying (Figure S8). The Pt L_3_‐edge XANES of Pt_1_Ni_1‐x_@Ni_x__NC exhibits the most intense white line among all samples, surpassing that of Pt_1_Ni_1_@NC and Pt foil, indicative of a substantially modified Pt surface electronic configuration (Figure 3c), in agreement with the XPS analysis (Figure 3a). A positive shift in the second‐derivative spectrum (Figure S9) further points to a higher density of unoccupied Pt 5d states, arising from selective dealloying and enhanced metal–support interactions. This electronic redistribution modulates the surface Pt coordination environment and optimizes the adsorption behavior of oxygenated intermediates toward the Sabatier optimum, thereby promoting ORR kinetics [32]. At the Ni K‐edge (Figure 3d), the absorption energies of Pt_1_Ni_1‐x_@Ni_x__NC and Pt_1_Ni_1_@NC lie between those of Ni foil and NiO, accompanied by a slight negative shift, suggesting an increase in the Ni oxidation state and strong Pt–Ni electronic coupling following dealloying. EXAFS analysis of the Pt L_3_‐edge (Figure 3e) reveals a contraction of the Pt–metal bond length (around 2.64 Å) relative to Pt foil (2.72 Å), arising from lattice compression upon Ni incorporation and additional surface stress induced by Ni segregation after dealloying.

Changes in the Ni K‐edge EXAFS are even more pronounced (Figure 3f). Both Pt_1_Ni_1‐x_@Ni_x__NC and Pt_1_Ni_1_@NC display markedly diminished Ni–Ni and Ni–O features compared to standards, alongside the emergence of a broad feature at 1.6 Å, attributable to Ni–N/C coordination formed post‐dealloying. The formation of Ni–N/C moieties is governed by a combination of thermodynamic preference and kinetic confinement. Thermodynamically, nitrogen functionalities exhibit strong coordination affinity toward Ni species, whereas kinetically, the released Ni is rapidly immobilized by abundant N sites [33], effectively suppressing long‐range diffusion and re‐alloying with Pt. This interpretation is corroborated by the atomically dispersed Ni distribution observed in aberration‐corrected electron microscopy. EXAFS fitting of the Pt L_3_‐edge (Figures S10, S11; Table S4) shows a reduced Pt–Pt coordination number, clear evidence of Pt–Ni bonding, and additional Pt–C/N/O contributions arising from interaction with the NC support. Transmission‐mode XAS further reveals decreased Pt–Pt and Pt–Ni coordination numbers and increased Pt–C/N/O coordination, mirroring the intensified Pt white‐line feature. Notably, Ni K‐edge fitting (Figures S12, S13; Table S5) confirms highly dispersed Ni atoms with negligible Ni–Ni coordination and enhanced Ni–C/N/O bonding, the latter shorter than typical Ni–O distances, reflecting strong metal–support interactions and Ni release during dealloying. Wavelet‐transform k‐space maps (Figures 3g–i and S14) vividly capture the coordination evolution of Pt and Ni through alloying and dealloying. The coordination evolution revealed by EXAFS provides direct insight into the enhanced durability of Pt_1_Ni_1__−__x_@Ni_x__NC.

**FIGURE 4:**
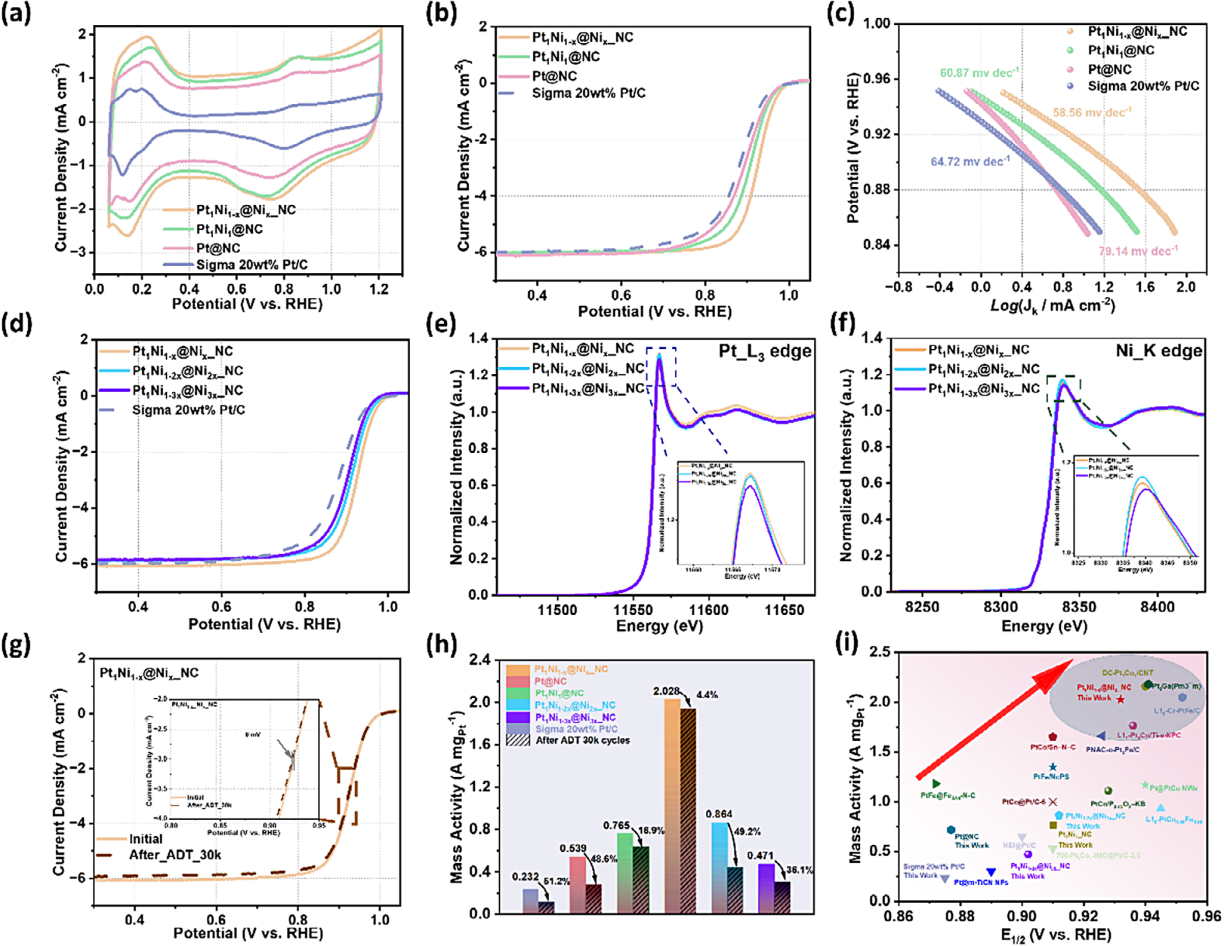
(a) N_2_‐saturated CV curves, (b) O_2_‐saturated ORR polarization curves, (c) Tafel slope of Pt@NC, Pt_1_Ni_1_@NC, Pt_1_Ni_1‐x_@Ni_x__NC, and Sigma 20wt% Pt/C. (d) O_2_‐saturated ORR polarization curves of Pt_1_Ni_1‐x_@Ni_x__NC, Pt_1_Ni_2‐x_@Ni_2x__NC, Pt_1_Ni_1‐3x_@Ni_3x__NC, and Sigma 20wt% Pt/C. (e) Pt_L_3_ edge XANES (inset: enlarged absorption near edge peak of Pt_L_3_), (f) Ni_K edge XANES (inset: enlarged absorption near edge peak of Ni_K) of Pt_1_Ni_1‐x_@Ni_x__NC, Pt_1_Ni_2‐x_@Ni_2x__NC, and Pt_1_Ni_1‐3x_@Ni_3x__NC. (g) ORR polarization curves before and after 30k ADT cycles of Pt_1_Ni_1‐x_@Ni_x__NC (inset: enlarged half‐wave overpotential position). (h) mass activity at 0.9 V versus RHE of all samples. (i) Mass activity and half‐wave overpotential comparison among electrocatalysts in this work and other recently reported Pt‐based electrocatalysts. All electrochemical tests were carried out in 0.1 M HClO_4_.

ORR measurements were performed to evaluate how coordination structure evolution and electronic optimization induced by alloying–dealloying influence catalytic performance. Cyclic voltammetry (CV) in N_2_‐saturated 0.1 M HClO_4_ (Figure 4a) shows that the MOF‐derived NC possesses a larger electrochemically active surface area and double‐layer capacitance than commercial Vulcan carbon. Among all catalysts, Pt_1_Ni_1‐x_@Ni_x__NC exhibits the most pronounced double‐layer feature, reflecting enhanced interfacial electron exchange. Correspondingly, ORR polarization curves (Figures 4b, S15) reveal that Pt_1_Ni_1‐x_@Ni_x__NC achieves the highest half‐wave potential (*E*_1/2_ at 0.932 V), outperforming Pt_1_Ni_1_@NC (0.910 V), Pt@NC (0.887 V), and commercial Sigma 20wt% Pt/C (0.875 V). The Tafel slope (Figure 4c) further confirms the accelerated kinetics, with Pt_1_Ni_1‐x_@Ni_x__NC exhibiting the lowest value (58.6 mV dec^−1^), consistent with its superior turnover frequency (Figure S16). These results demonstrate that structural evolution during dealloying optimizes ORR kinetics and enhances catalytic activity. To further probe the structure–activity relationship, Pt_1_Ni_1‐2x_@Ni_2x__NC and Pt_1_Ni_1‐3x_@Ni_3x__NC were synthesized for comparison. As shown in Figures 4d and S17–S19, their *E*_1/2_ values (0.912 and 0.902 V, respectively) and Tafel slopes (58.9 and 59.6 mV dec^−^^1^, respectively) are inferior to Pt_1_Ni_1‐x_@Ni_x__NC, underscoring the unique advantage of its optimized coordination environment in boosting ORR activity. Consistently, Pt_1_Ni_1‐x_@Ni_x__NC also exhibits a higher turnover frequency, indicating intrinsically enhanced reaction kinetics at the active Pt sites. Further XPS analysis (Figure S20 and Tables S6, S7) reveals that with increasing dealloying temperature, the binding energies of Pt^0^ and Pt^2^^+^ in Pt_1_Ni_1‐2x_@Ni_2x__NC (71.98/75.30 eV and 73.14/76.46 eV) and Pt_1_Ni_1‐3x_@Ni_3x__NC (71.71/75.03 eV and 72.87/76.19 eV) shift to lower values compared to those in Pt_1_Ni_1‐x_@Ni_x__NC (72.10/75.42 eV and 73.26/76.58 eV), while the corresponding Ni 2p binding energies increase. This trend indicates that elevated dealloying temperatures alter the surface chemical states of Pt and Ni and weaken their intermetallic electronic interactions. To further probe these effects, XAS was employed to analyze the structural and coordination evolution. As shown in Figure 4e,f, the Pt L_3_‐edge white‐line intensity gradually decreases with increasing dealloying temperature, whereas the Ni K‐edge intensity first increases and then decreases. Such behavior likely arises from further dissolution of Ni atoms followed by their partial reaggregation on the support. This interpretation is supported by EXAFS analysis (Figures S21–S23; Tables S8, S9), which shows that the Pt–Pt coordination number increases in Pt_1_Ni_1‐2x_@Ni_2x__NC (5.4) and Pt_1_Ni_1‐3x_@Ni_3x__NC (5.6) compared with Pt_1_Ni_1‐x_@Ni_x__NC (5.3), while the Pt–Ni coordination number decreases, confirming the progressive release of Ni atoms from the PtNi alloy at higher dealloying temperatures. Meanwhile, the Ni–Ni coordination number first decreases and then increases, whereas the Ni–C/N/O coordination number exhibits the opposite trend, consistent with the reaggregation of Ni atoms onto the support, as also reflected in the Ni K‐edge features (Figure 4f).

Accelerated durability testing (ADT, Figure S24) demonstrated that Pt_1_Ni_1‐x_@Ni_x__NC exhibits superior catalytic stability. Its ORR polarization curve remained essentially unchanged after 30,000 cycles, with a half‐wave potential loss of only 6 mV (Figure 4g). Normalized ORR activities further highlight its outstanding performance, Pt_1_Ni_1‐x_@Ni_x__NC achieves a mass activity of 2.028 A mg_Pt_^−^^1^, surpassing Pt_1_Ni_1_@NC, Pt@NC, Pt_1_Ni_1‐2x_@Ni_2x__NC, Pt_1_Ni_1‐3x_@Ni_3x__NC, and Sigma 20wt% Pt/C by factors of 2.65, 2.82, 2.35, 4.31, and 8.75, respectively (Tables S10–S11, Figure 4h). Importantly, the mass activity decays by only 4.4% after ADT, underscoring its remarkable durability (Figure S25). The enhanced activity and stability of Pt_1_Ni_1‐x_@Ni_x__NC can be attributed to strengthened Pt–Ni intermetallic interactions arising from coordination restructuring under optimized dealloying conditions, while stabilized Pt coordination suppresses atomic rearrangement and particle coarsening. In parallel, immobilization of released Ni as Ni–N/C moieties block dissolution–migration pathways, collectively accounting for the excellent long‐term stability (Tables S4, S5, S8, and S9). Compared with state‐of‐the‐art catalysts, Pt_1_Ni_1‐x_@Ni_x__NC occupies a leading position in ORR performance (Figure 4i).

**FIGURE 5:**
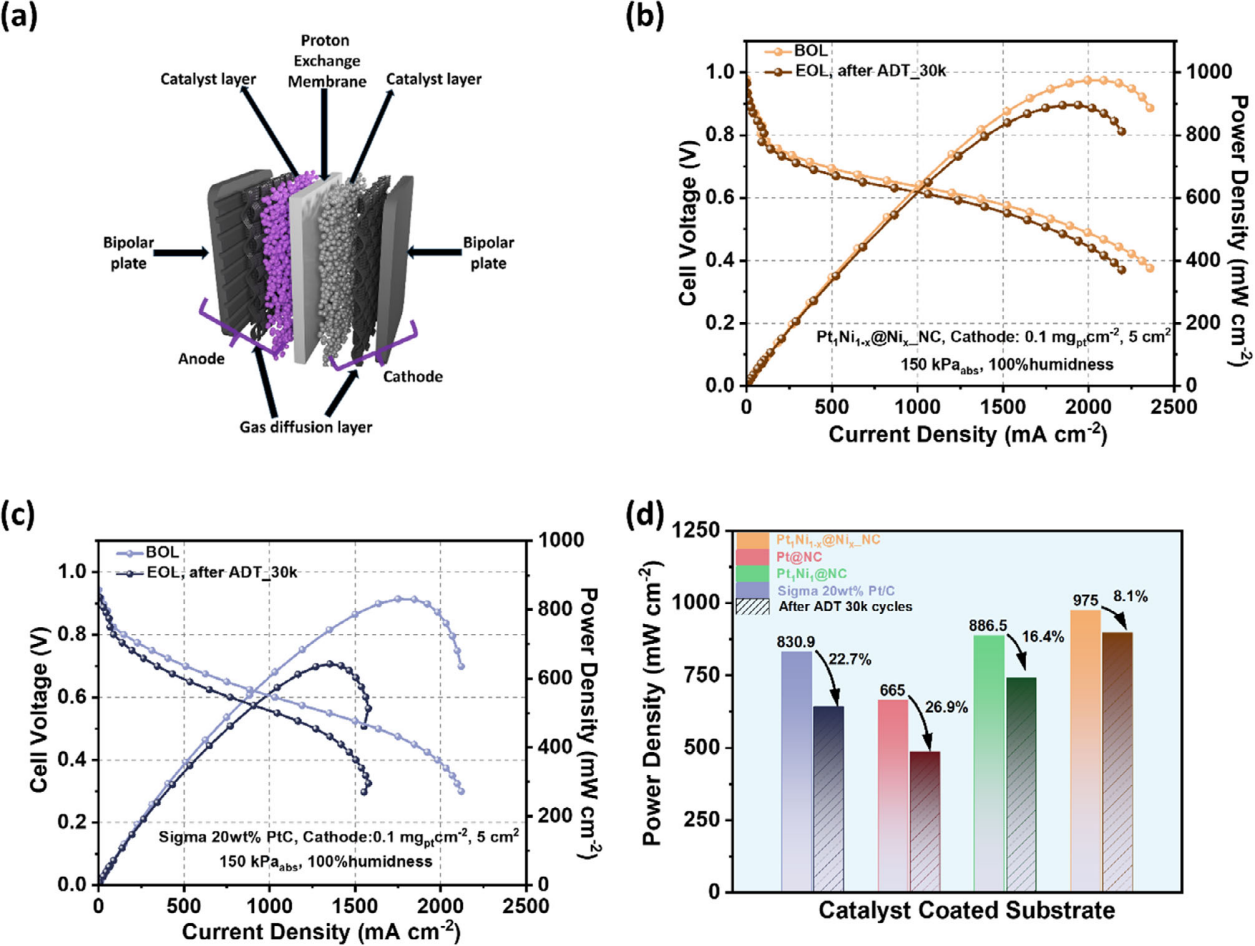
(a) Schematic illustration of the structural configuration of a fuel cell stack. (b) MEA performance of Pt_1_Ni_1‐x_@Ni_x__NC cathode before (BOL) and after ADT (EOL) under the H_2_/air condition. (c) MEA performance of Sigma 20 wt% Pt/C cathode before (BOL) and after ADT (EOL) under the H_2_/air condition. (d) Peak power density of all tested MEA before and after ADT.

While RDE measurements reflect intrinsic ORR kinetics under idealized mass‐transport conditions, MEA performance is additionally governed by catalyst–ionomer interactions, gas diffusion, and water management within the electrode architecture. Further, the catalysts’ membrane electrode assembly (MEA) performance was assessed under practical fuel cell operating conditions (cathode catalyst loading: 0.1 mg_Pt_ cm^−2^, 80°C, 150 kPa_abs_ backpressure; Figures 5a and S26). Humidity‐dependent MEA tests revealed excellent tolerance: the peak power density increased from 908 mW cm^−2^ at 60% relative humidity to 975 mW cm^−2^ at 100% (Figure S27). Pt_1_Ni_1‐x_@Ni_x__NC also demonstrated remarkable cycling stability (Figure 5b), maintaining high performance without significant water flooding after 30,000 cycles, unlike commercial Pt/C (Figure 5c). This advantage is attributed to the present system; the Pt‐rich shell ensures high intrinsic ORR activity and minimizes kinetic losses, thereby extending the operational regime toward mass‐transport‐limited conditions. Concurrently, the N‐doped carbon framework and Ni–N/C moieties create polar interfacial domains and an interconnected porous architecture that promote efficient water removal and oxygen diffusion while preserving effective ionomer–catalyst contact. Together, these synergistic features alleviate transport‐related limitations and underpin the high peak power density and stable performance at high current densities observed for Pt_1_Ni_1‐x_@Ni_x__NC. Furthermore, Figure 5d illustrates that the peak power density retention from beginning‐of‐life (BOL) to end‐of‐life (EOL) was 91.9% (8.1% loss, Figure S28) for Pt_1_Ni_1‐x_@Ni_x__NC, substantially outperforming Pt/C (22.7% loss, Figure S29), Pt@NC (26.7% loss, Figure S30), and Pt_1_Ni_1_@NC (16.4% loss, Figure S31). Notably, the achieved peak power density and durability rank among one of the highest reported for Pt‐based catalysts under comparable MEA testing conditions (Table S12). These superior performances underscore the effectiveness of the controllable alloying–dealloying and confinement strategy, which enables a robust Pt‐rich shell/ordered‐alloy core configuration coupled with Ni–N/C interfacial sites, collectively stabilizing active centers and optimizing water management for long‐term PEMFC operation.

## 3 Conclusion

In summary, we establish a controllable alloying–dealloying strategy to construct PtNi nanoparticles confined within an N‐doped carbon framework, yielding a Pt_1_Ni_1‐x_@Ni_x__NC catalyst with a Pt‐rich shell and stabilized Ni_NC structure. Ex‐situ spectroscopic analyses reveal that selective dealloying tailors the Pt coordination environment and electronic structure, while the NC support reincorporates dissolved Ni, thereby suppressing agglomeration and enhancing durability. Consequently, Pt_1_Ni_1‐x_@Ni_x__NC delivers exceptional ORR activity (*E*_1/2_ = 0.932 V, mass activity 2.028 A mgPt^−^^1^) and stability, with only a 6 mV loss in half‐wave potential after 30,000 cycles. Beyond RDE tests, the catalyst demonstrates outstanding MEA performance under practical fuel‐cell operating conditions (cathode catalyst loading: 0.1 mg_Pt_ cm^−2^, 80°C, 150 kPa_abs_ backpressure). Pt_1_Ni_1‐x_@Ni_x__NC achieves a peak power density of 975 mW cm^−2^ at 100% RH and exhibits remarkable cycling durability, retaining 91.9% of its beginning‐of‐life power density after 30,000 cycles, significantly outperforming commercial Pt/C and other PtNi benchmarks. In this work, the superior RDE activity of Pt_1_Ni_1‐x_@Ni_x__NC, arising from its Pt‐rich shell and optimized electronic structure, translates into reduced kinetic losses in MEA. Meanwhile, the N‐doped carbon confinement and Ni–N/C moieties promote effective ionomer coverage, facilitate water evacuation, and maintain open gas‐transport pathways. This synergy enables the intrinsic activity advantages observed in RDE tests to be preserved under practical PEMFC operating conditions.

Importantly, these results place Pt_1_Ni_1‐x_@Ni_x__NC among the leading candidates for practical fuel‐cell applications. Unlike conventional PtNi alloying or dealloying strategies that often suffer from Ni dissolution and nanoparticle coarsening under MEA conditions, the present alloying–dealloying–support coupling strategy resolves the long‐standing trade‐off between activity and stability. By simultaneously forming a Pt‐rich shell and immobilizing released Ni as Ni–N/C moieties, a dual stabilization mechanism is established that preserves the optimized Pt electronic structure while effectively suppressing degradation pathways relevant to practical PEMFC operation. Looking forward, integrating such coordination and support‐engineering strategies with advanced intermetallic ordering or dual‐metal‐site carbon supports may further mitigate transition‐metal leaching and unlock the durability required for fuel cells and beyond.

## Conflicts of Interest

The authors declare no conflicts of interest.

## Supporting information




**Supporting File 1**: anie71482‐sup‐0001‐SuppMat.docx.

## Data Availability

The data that support the findings of this study are available on request from the corresponding author. The data are not publicly available due to privacy or ethical restrictions.
