# Supplementary material for: Harnessing Controlled Dealloying–Support Coupling for Ultrastable PtNi Catalysts in PEMFC Applications
Source: Angew Chem Int Ed Engl. 2026 Feb 9;65(12):e4524344. doi: 10.1002/anie.4524344 (PMC12990961; doi:10.1002/anie.4524344)
Supplement: Supplementary file 1 — Supporting File 1: anie71482‐sup‐0001‐SuppMat.docx. [file ANIE-65-e4524344-s001.docx]

**Harnessing Controlled Dealloying–Support Coupling for Ultrastable PtNi Catalysts in PEMFC Applications**

Fei Guo^a^†, Manxi Gong^a^†, Longxiang Liu^b^, Bochen Li^c^, Ruwei Chen^a^, Mengjun Gong^d^, Wei Zong^a^, Jianuo Chen^c^, Qi Li^e^, Jing Li^e^, Yunpeng Zhong^a^, Zeyi Zhang^f^, Jianrui Feng^a^, Rhodri Jervis^c^, Guanjie He^a^*

^a^ Department of Chemistry, University College London, London, WC1H 0AJ UK

^b^ Department of Materials, University of Oxford, Parks Road, Oxford OX1 3PH, U.K.

^c^ Electrochemical Innovation Lab, Department of Chemical Engineering, University College London, London WC1E 7JE, UK

^d^ Department of Chemistry, Imperial College London, White City Campus, London W12 0BZ, United Kingdom

^e^ Department of Chemistry, School of Physical and Chemical Sciences, Queen Mary University of London, London, E1 4NS UK

^f^ Department of Chemistry, University of Zurich, Winterthurerstrasse 190, CH-8057 Zurich, Switzerland

**Experimental Section:**

**Chemicals and materials:** All the chemicals used were commercial without any further purification. Zinc nitrate hexahydrate (Zn(NO3)2·6H2O; >99.0%), 2-methyleimidazole (99.0%), methanol (99.0%), Nickel(II) chloride hexahydrate (NiCl_2_·6H_2_O; >97.0%), ethanol (99.0%), Chloroplatinic acid solution (H_2_PtCl_6_; 8 wt.% in H_2_O), Commercial platinum on carbon (Com. 20 wt% PtC, 20 wt.%Pt), sulfuric acid (H_2_SO_4_, 95%), Perchloric acid (HClO_4_, 70-72%), Cellulose (microcrystalline, powder, 20 μm), all these chemicals were purchased from Sigma-Aldrich (UK) Co., Ltd, and de-ionized water with the specific resistance of 18.25 MΩ·cm^-1^ (obtained by reversed osmosis followed by ion-exchange and filtration).

**Materials synthesis**

**Preparation of NC (Nitrogen doped Carbon derived from ZIF-8):** The NC was prepared according to a reported method (with 20 mmol Zn(NO_3_)_2_·6H_2_O, 82.58 mmol 2-methyleimidazole). The prepared ZIF-8 was annealed at 1050℃ for two hours under N_2_ atmosphere. Then the powder was washed with 0.5 M H_2_SO_4_ for 12 hours, denoted as NC.

**Synthesis of Pt_1_Ni_1_@NC:** The H_2_PtCl_6_ (0.05 mol) and NiCl_2_·6H_2_O (0.05 mol) were mixed in 10 ml ethanol, then added the pre-prepared NC (50 mg) into those two solution stirring for five hours, the sample was washed by ethanol, then annealed at 750℃ for 2h in a 5% H_2_/95% N_2_ atmosphere. The prepared sample was denoted as Pt_1_Ni_1_@NC.

**Synthesis of Pt_1_Ni_1-n_@Ni_n__NC:** The prepared Pt_1_Ni_1_@NC was then further annealed at 100℃, 200℃, 300℃ under ammonia (NH_3_) atmosphere. The further annealed samples were denoted as Pt_1_Ni_1-x_@Ni_x__NC, Pt_1_Ni_1-2x_@Ni_2x__NC, Pt_1_Ni_1-3x_@Ni_3x__NC respectively (n=x, 2x, 3x referring to temperature 100℃, 200℃, 300℃)

**Synthesis of Pt@NC:** The H_2_PtCl_6_ (0.05 mol) was dissolved in 10 ml ethanol, then added the pre-prepared NC (50 mg) into those two solution stirring for five hours, the sample was washed by ethanol, then annealed at 750℃ for 2h in a 5% H_2_/95% N_2_ atmosphere. The prepared sample was denoted as Pt@NC.

**Materials characterization**

The structure and morphology of the samples were observed on scanning electron microscope (SEM, JEOL 7600F), transmission electron microscopy (JEOL, JEM-2100), and scanning transmission electron microscopy (JEOL ARM200CF (E01) and JEOL ARM300C (E02) of electron Physical Science Imaging Centre (ePSIC) from Diamond Light Source (UK)). The XRD patterns were performed on a PANalytical Empyrean device with Cu Kα radiation. X-ray photoelectron spectroscopy (XPS, Thermo scientific K‐alpha photoelectron spectrometer) was conducted to determine the chemical state of surface elements. The X-ray absorption spectroscopy (XAS) experiments were performed at beamline B18 of Diamond Light Source (UK). The schematic illustration of the beamline end-station set-up of B18@Diamond Light Source is displayed in **Figure S30**. Data was collected by transmission model. The samples were prepared by grinding them with cellulose then pressing them into pallets (diameter 8mm, **Figure S30**). 3 repetitions of XAS scans were collected and averaged to improve the signal-to-noise ratio for both Pt_L edge and Ni_K edge of each sample. The energy shift was calibrated based on the feature of incident beam current (I0). Arctangent step function and Gaussian peak functions were used to perform peak fitting on Athena software. Inductively coupled plasma mass spectrometer (ICP-MS) (iCAP7000, Thermo Fisher Scientific) was used to determine the Pt and Ni loading. The particle size distribution was determined by measuring more than 200 particles using a Nano Measurer.

**Electrochemical Tests**

All the electrochemical tests were carried out in a three-electrode cell system controlled by a Gamry potentiostat. The RDE working electrode comprises a glassy carbon rotation disk electrode (0.19625 cm2 area). An Ag/AgCl electrode and a graphite rod were used as the reference electrode and the counter electrode, respectively. 0.3 μm, 0.1 μm, and 0.05 μm Al_2_O_3_ polishing suspension were successively used to clean the working electrode. To prepare the ink, 2.0 mg of catalyst was dispersed in 980 uL (ethanol and isopropanol, volume ratio at 1:1) and 20 μL of Nafion (5 wt%, D520) solution. After ultrasonication for 45 min, 10.0 μL suspension was drop-casted onto the disk electrode on the spinning stage and dried at room temperature. The oxygen reduction reaction (ORR) activity of the samples was evaluated by RDE measurements in 0.1 M HClO_4_. Cyclic voltammetry (CV) was first performed at a scan rate of 50 mV s^-1^ between 0.05 and 1.2 V in N_2_-saturated electrolytes at room temperature until a steady CV was obtained. After that, the ORR activity was measured in 0.1 M HClO_4_ saturated with O_2_ at 1600 rpm using positive-going linear sweep voltammetry (LSV) at a scan rate of 2 mV s^−1^. The electrochemical impedance spectroscopy (EIS) was measured for iR correction. Note that all the LSV tests were 95% iR-compensated. The accelerated durability test (ADT) was applied to evaluate catalyst stability by cycling the potentials ranging from 0.6 to 0.95 V at a scan rate of 100 mV s^−1^ at room temperature in O_2_-saturated 0.1 M HClO4.

All electrode potentials were calibrated to RHE using the Eq. (1):

$E_{RHE}= E_{\frac{Ag}{Agcl}}+0.197 V+0.059 pH-0.95iR$ (1)

The Tafel slopes were calculated by fitting to the Tafel Eq. (2):

$\eta= b\log j+c$ (2)

The TOF values were calculated based on the Pt atom numbers in each sample electrode according to the following Eq. (3):

$TOF= \frac{I}{2Fn}$ (3)

where I (in Amperes) is the current measured from LSV curves, F is the Faraday constant (96485.3, in C mol^-1^) and n represents the atom number of Pt (in mol).

**Fuel Cell Tests**

The synthesized catalysts were incorporated into the membrane electrode assembly (MEA) using a 15 μm-thick Gore membrane with an active area of 5 cm². To prepare the catalyst ink, the catalysts were dispersed in a mixture of deionized water and isopropanol (volume ratio 3:7), along with a Nafion ionomer (5 wt%, D520, I/C ratio of 0.7), and subjected to ultrasonication for 45 minutes. The resulting ink was directly sprayed onto a gas diffusion electrode (GDE; Freudenberg H24C × 483, PTFE-treated, 0.25 mm thickness, with a microporous layer) to achieve a cathode Pt loading of 0.1 mg_Pt cm^-2^. Commercial 20 wt% Pt/C was used as the anode catalyst, with a Pt loading of 0.025 mg_Pt cm^-2^. The MEA was assembled by sandwiching it between two graphite bipolar plates, H_2_-air fuel cell testing was carried out in a single cell using 850e Multi-Range Fuel Cell Test System (Scribner, LLC.), as shown in **Figure S31**. A 12-hour break-in process was performed by three steps: 1. Heating up the whole cell to 80 ℃; 2. purge N_2_/N_2_ at the flow rates of 200 standard cubic centimeters per minute (sccm) at the same temperature to add humidity to 100%; 3. then the anode and cathode were purged with H_2_ and air with flow rates of 200 and 200 sccm and applying voltage scans between 0.9 and 0.3 V with a 50-mV staircase for 30 minutes at each voltage. During the break-in procedure, the cell temperature was keeping at 80 ℃, the back pressure at 150 kPa_abs_, the humidity at 100 %. Then polarization test was used to measure the full cell performance, applying voltage scans between open circuit voltage (OCV) to 0.3 V with a 20-mV scan rate. During the polarization test, the cell was operated at 80 °C, with 150 kPa_abs_, the humidity at 100/90/80/70/60 %, H_2_/air, and a gas flow rate of 500/2000 sccm for anode/cathode, respectively. The accelerated potential cycling durability test (ADT) was conducted using the trapezoidal wave method from 0.6 to 0.95 V with 3 s rise time and 3 s hold time (150 kPa_abs_ H_2_/N_2_ 200/200 sccm, 80 °C, 100 % humidity).

**DFT method**

DFT calculations were performed in the Quantum Espresso (QE) v7.2^[1]^ electronic structure code, using the PBE exchange correlation functional. The projected augmented wave (PAW) pseudopotentials were used from the Pseudo-Dojo database^[2]^. A 32 Ry planewave cutoff and a 256 Ry density cutoff were used.3 × 3 × 1 Monkhorst-Pack k-point grids were for slab calculations. The convergency was tested for cutoff energy. For all the slab calculations, the Brillouin zone sampling was restricted to the Gamma point only. A Grimme type D3 correction was used for presenting van der Waals force. Spin-polarized calculations were performed. The criterion of SCF convergency was set to 1 × 10-6 Ry. All atomic positions were optimized using the Broyden-Fletcher-Goldfarb-Shanno (BFGS) algorithm. The criterion for geometry optimization was set to 3 × 10-3 Ry/Bohr. The free energy for each reaction intermediate is defined by Eq. (4):

ΔG = ΔEDFT + ΔEZPE – TΔS + eU (4)

ΔEDFT is the adsorption energy calculated by DFT. ΔEZPE is the zero-point energy change (ZPE) and ΔS is the entropy change at 300 K. T, e, and U are the temperature, the number of electrons transferred, and the electrode potential, respectively. The correction of entropy and ZPE items are taken from previous reports (+0.05 eV, +0.35 eV and +0.40 eV for O*, OH* and OOH*, respectively)^[3-4]^.

**
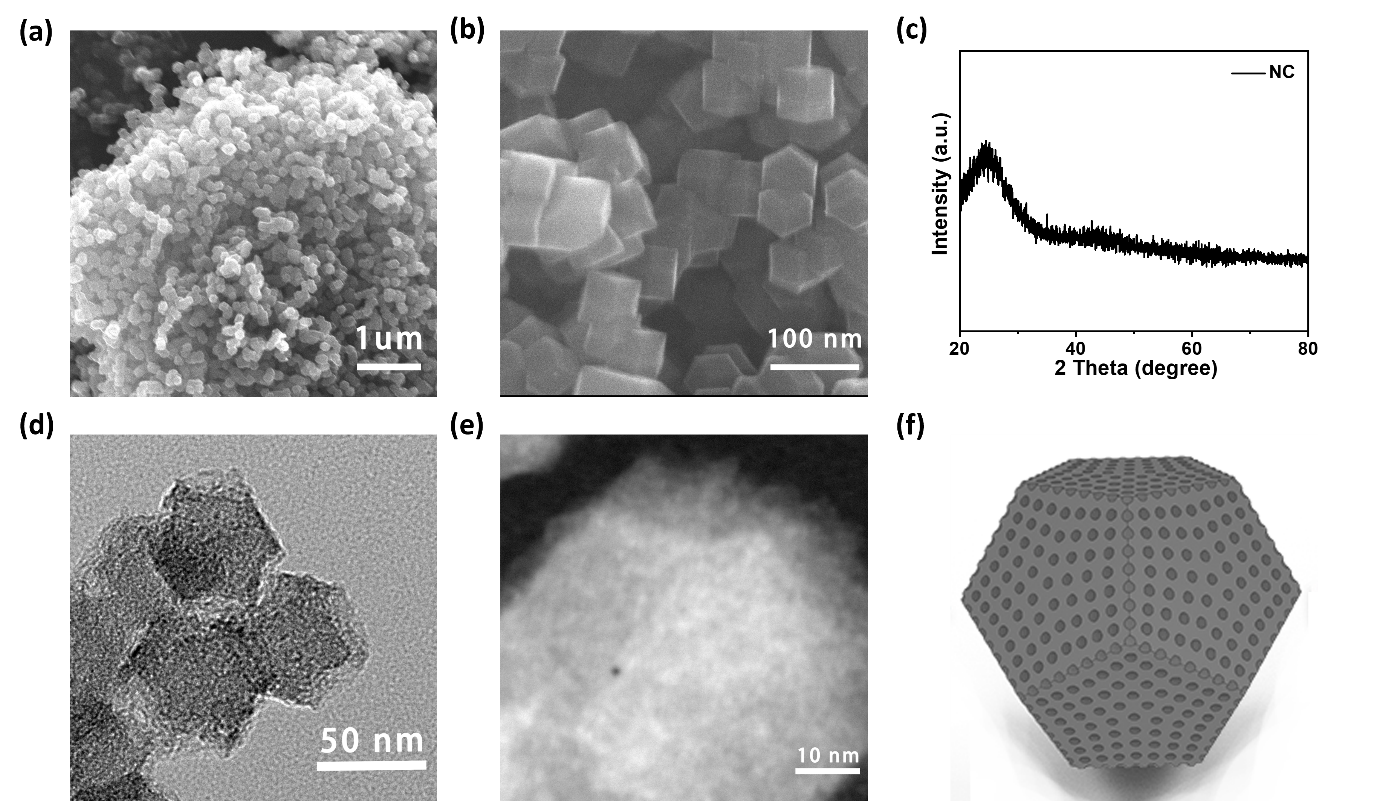
**

**Figure S1.** (a-b) SEM images of NC. (c) XRD pattern of NC. (d-e) BF TEM image and HAADF-STEM image of NC. (f) Structure diagram of NC.


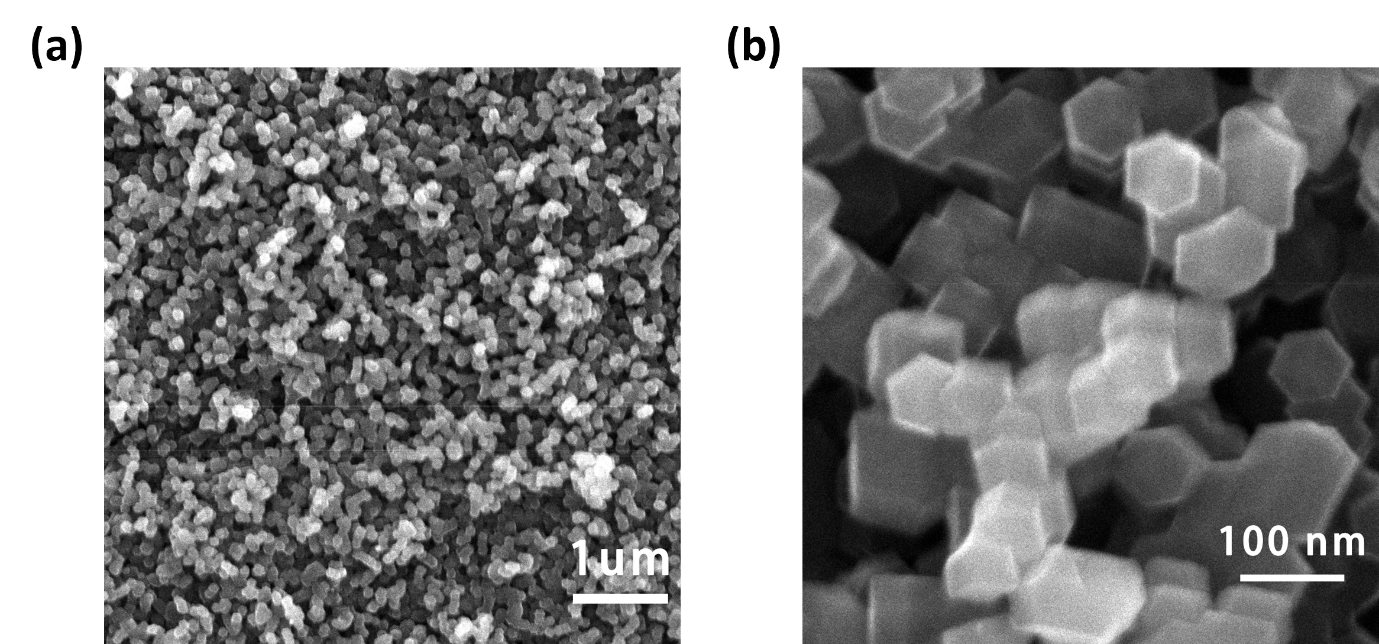


**Figure S2.** (a-b) SEM images of Pt_1_Ni_1-x_@Ni_x__NC.


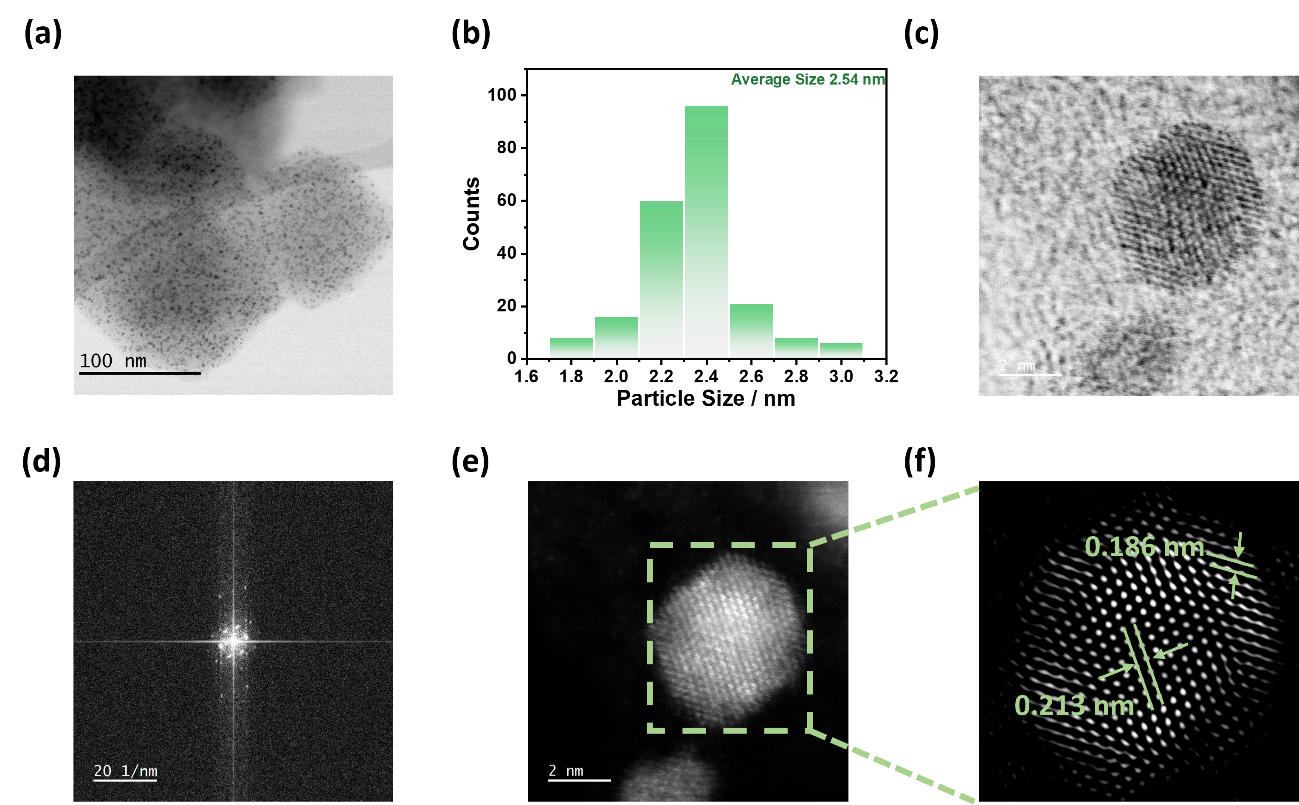


**Figure S3.** (a) BF-TEM image of Pt_1_Ni_1_@NC. (b) Size distribution of the nanoparticles from (a). (c) HRSTEM image of Pt_1_Ni_1_@NC. (d) FFT image from (c). (e) HADDF-STEM image of Pt_1_Ni_1_@NC. (f) Inverse FFT image from (e).

**
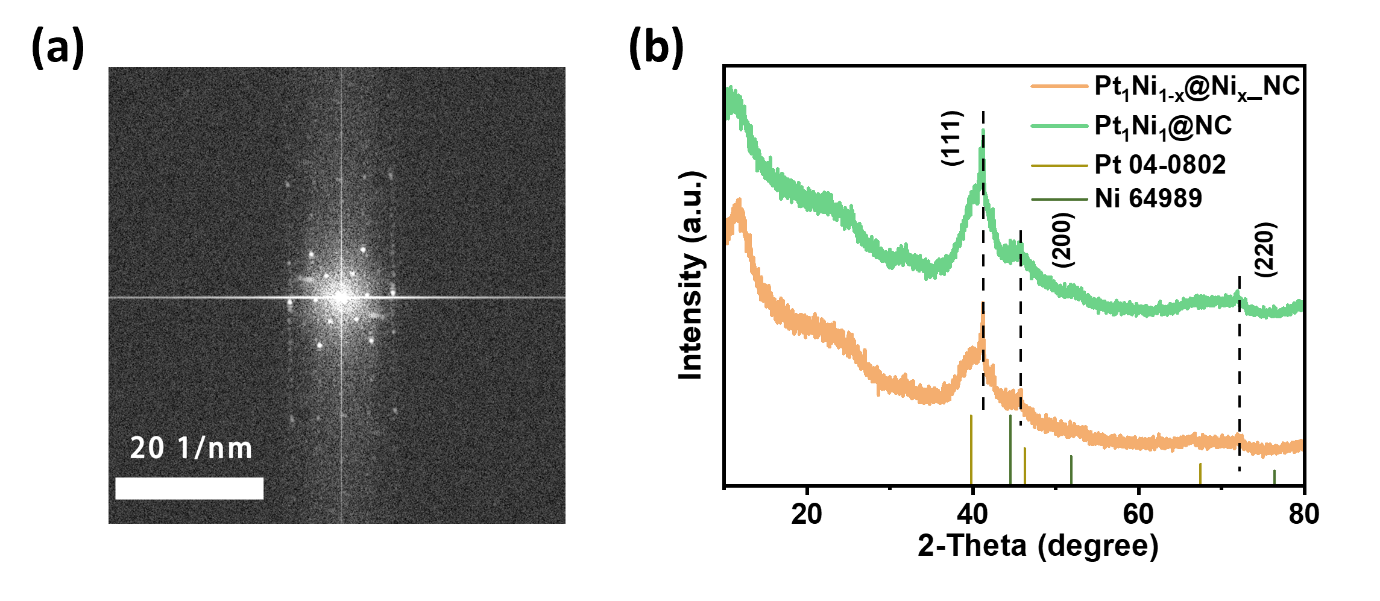
**

**Figure S4.** (a) FFT image of Pt_1_Ni_1-x_@Ni_x__NC from **Figure 2d**. (b) XRD patterns of Pt_1_Ni_1_@NC and Pt_1_Ni_1-x_@Ni_x__NC.

**
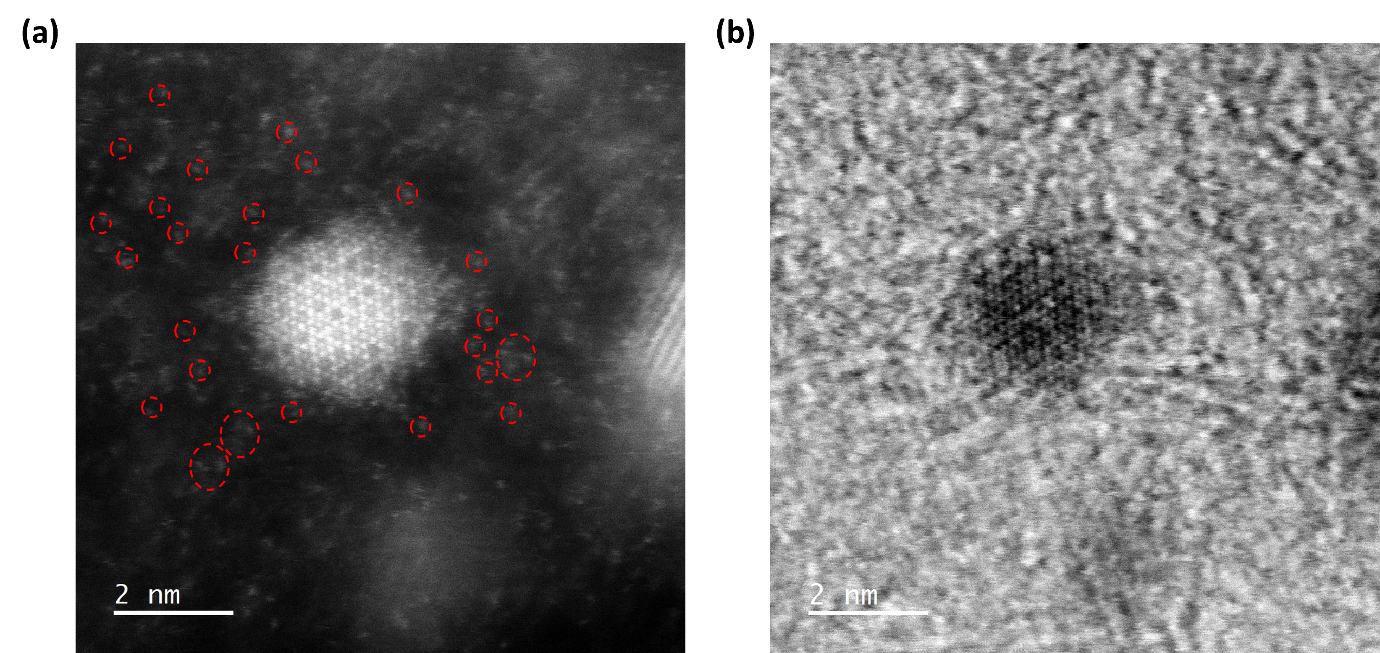
**

**Figure S5.** (a) HAADF-STEM image (The red circle highlights the bright contrast features surrounding the Pt_1_Ni_1-x_ nanoparticles) (b) BF-STEM image of Pt_1_Ni_1-x_@Ni_x__NC.

**
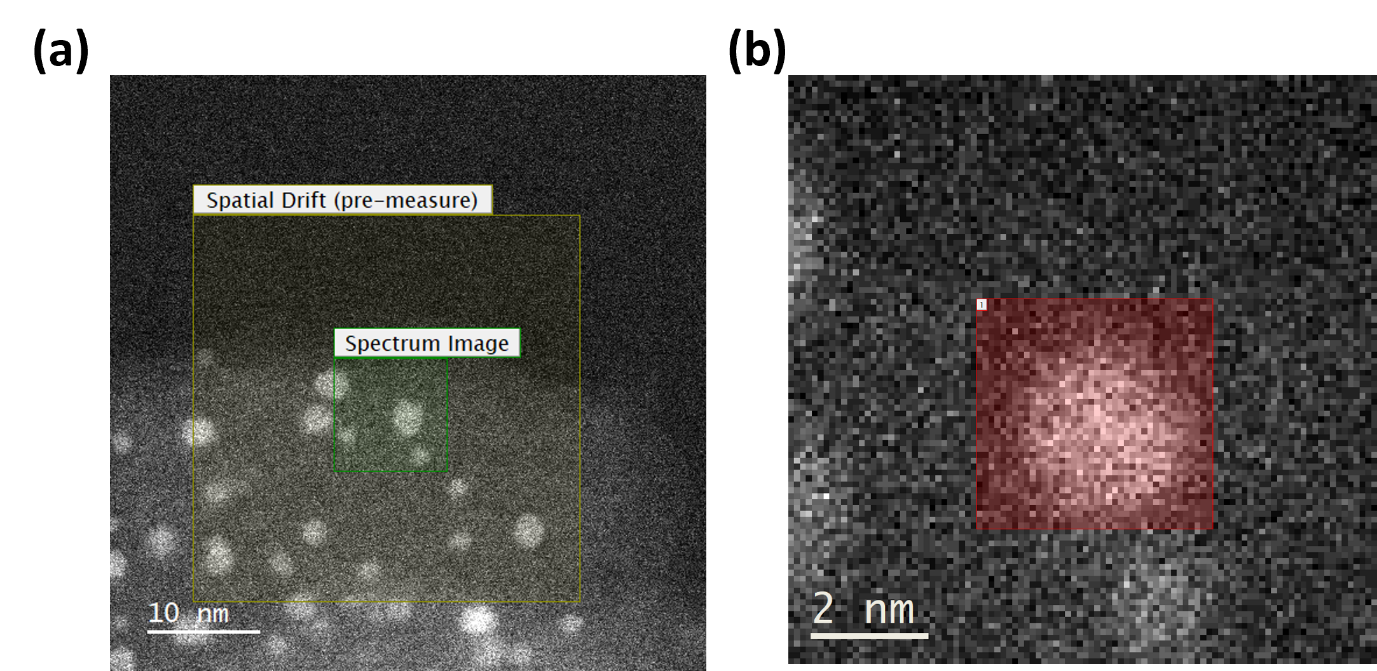
**

**Figure S6.** (a) Pre-measurement (large area, with the scale bar of 10 nm) for linear scan of Pt_1_Ni_1-x_@Ni_x__NC. (b) Pre-measurement (focused on one nanoparticle, with the scale bar of 2 nm) for linear scan of Pt_1_Ni_1-x_@Ni_x__NC.

**
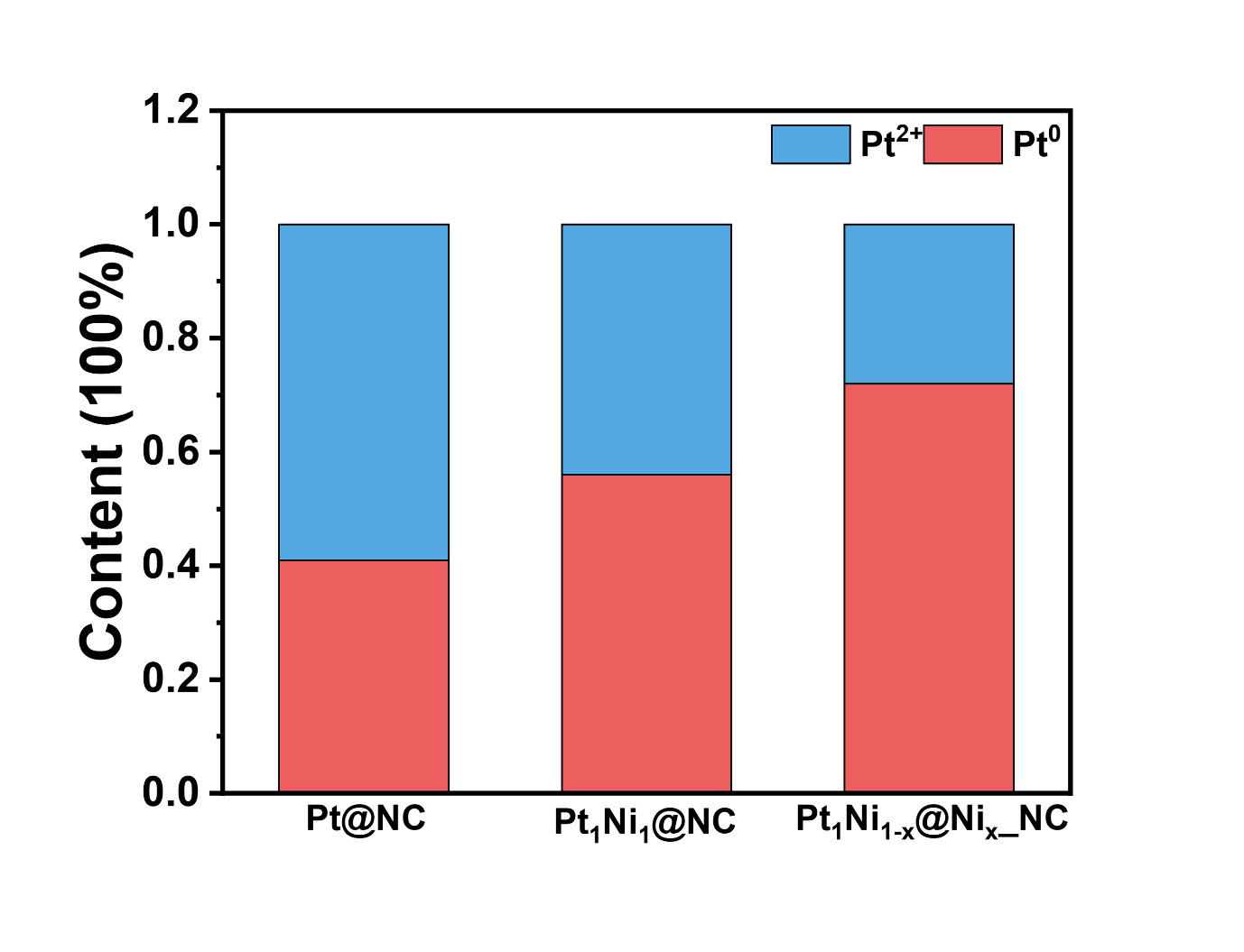
**

**Figure S7.** The content data of fitting results from Pt 4f xps spectra (**Figure 3a**).

**
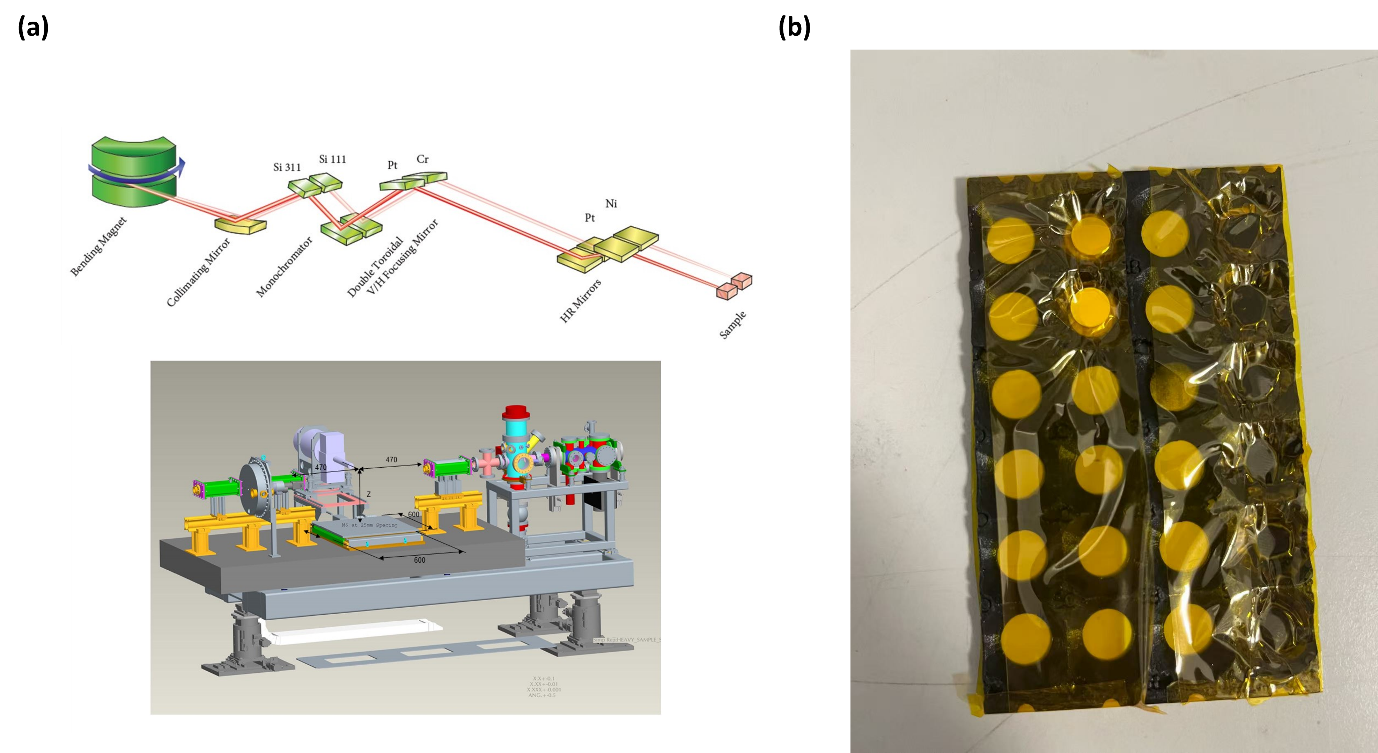
**

**Figure S8.** (a) Beamline Layout and Specifications (B18 in Diamond Light Source). (b) Prepared sample pallets for XAFs test.

**
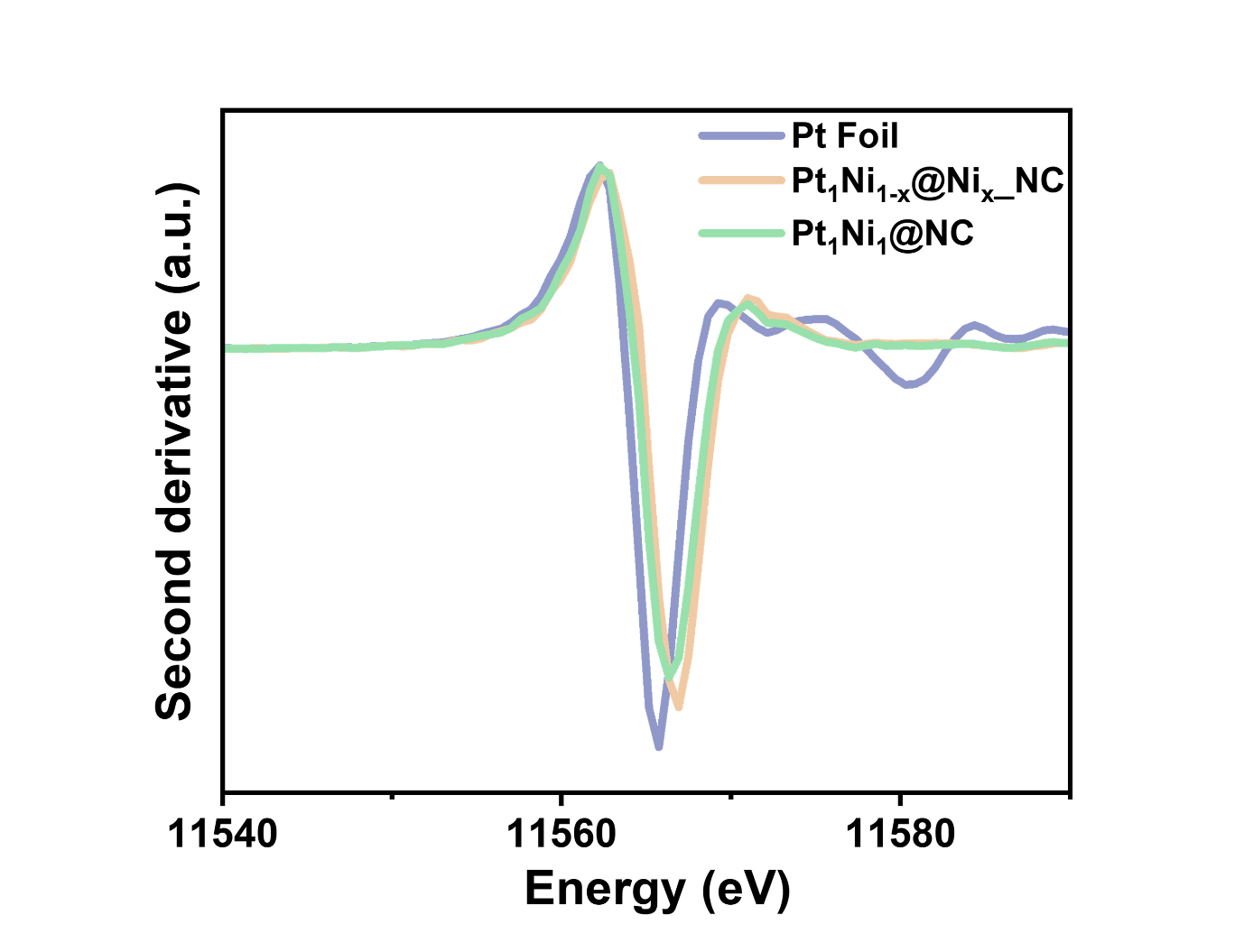
**

**Figure S9.** The second derivative of the Pt L3-edge XANES of Pt Foil, Pt_1_Ni_1_@NC and Pt_1_Ni_1-x_@Ni_x__NC.

**
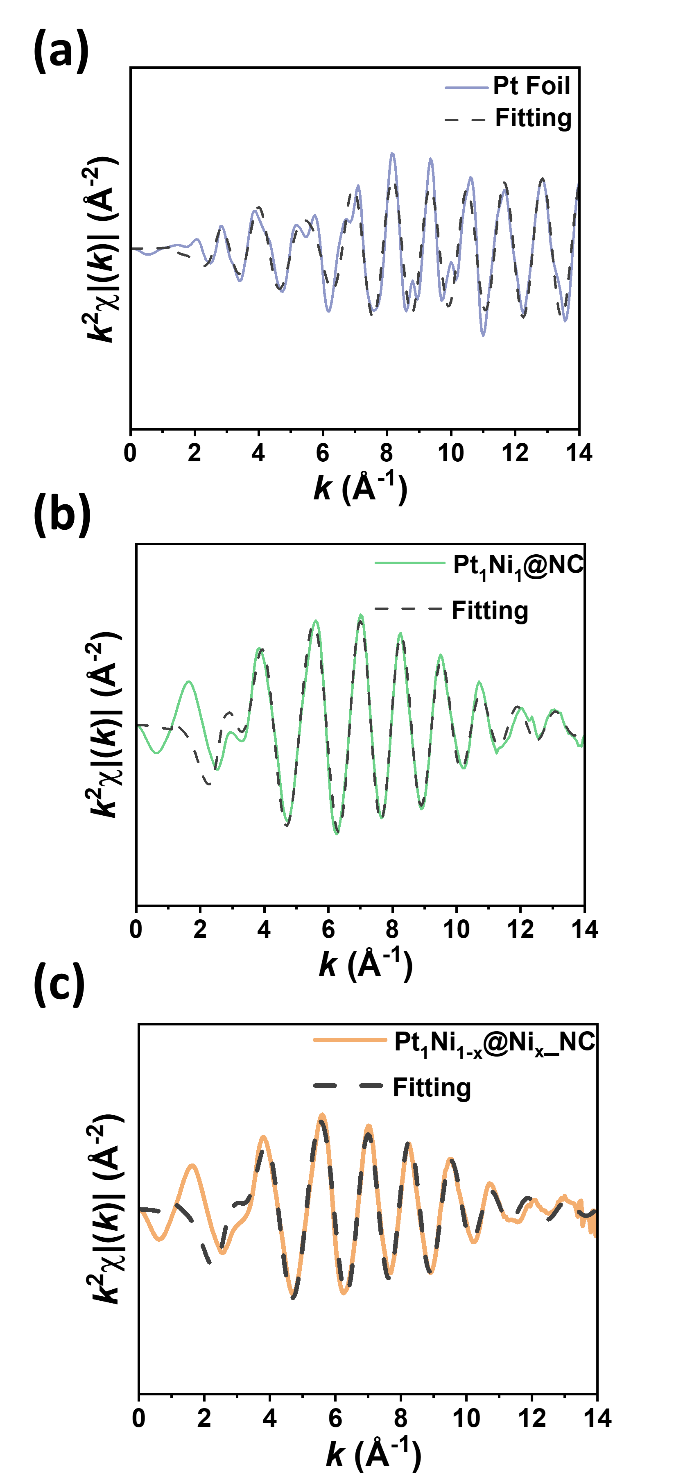
**

**Figure S10.** The experimental data (lines) and fitting data (dots) of Pt L_3_-edge EXAFS spectra for Pt Foil (a), Pt_1_Ni_1_@NC (b) and Pt_1_Ni_1-x_@Ni_x__NC (c), shown in k^2^-weighted K-space.

**
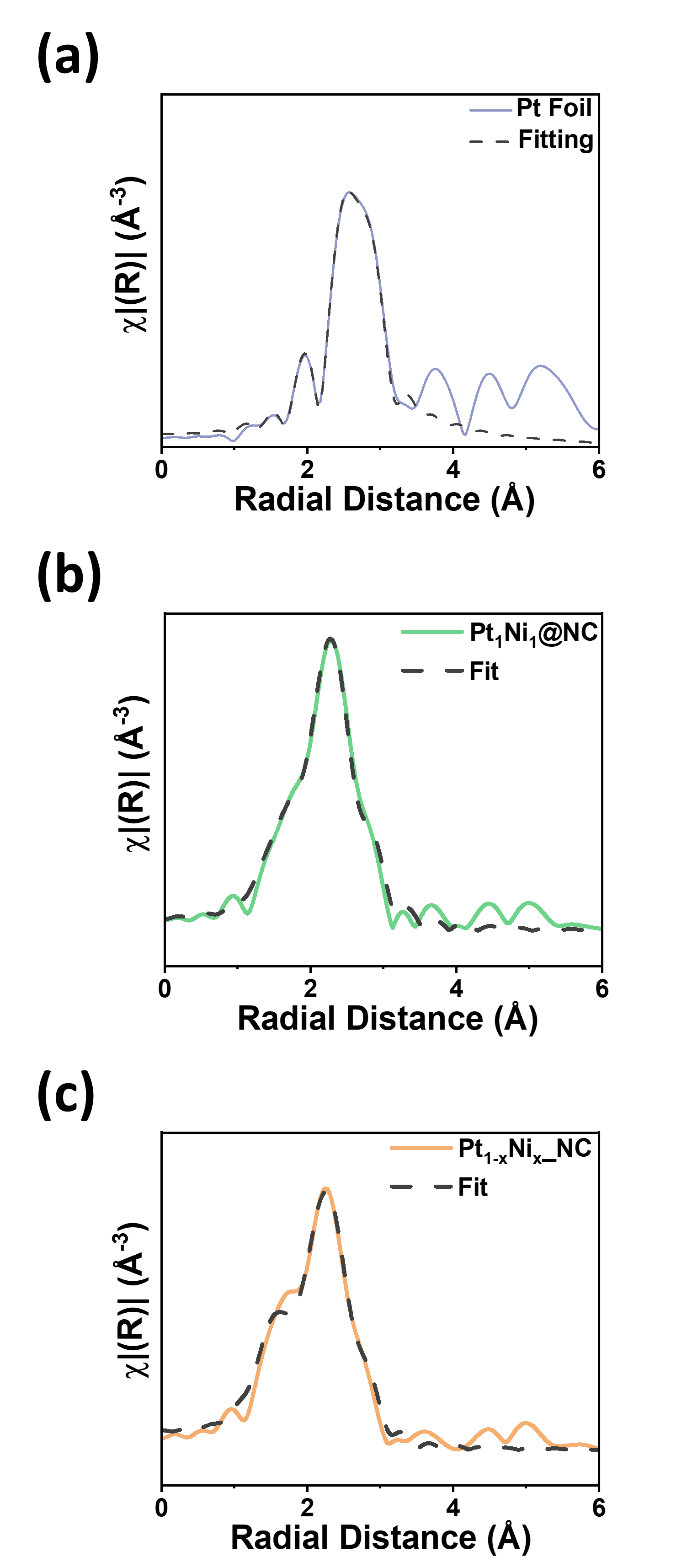
**

**Figure S11.** The experimental data (lines) and fitting data (dots) of Pt L_3_-edge EXAFS spectra for Pt Foil (a), Pt_1_Ni_1_@NC (b) and Pt_1_Ni_1-x_@Ni_x__NC (c), shown in k^2^-weighted R-space.

**
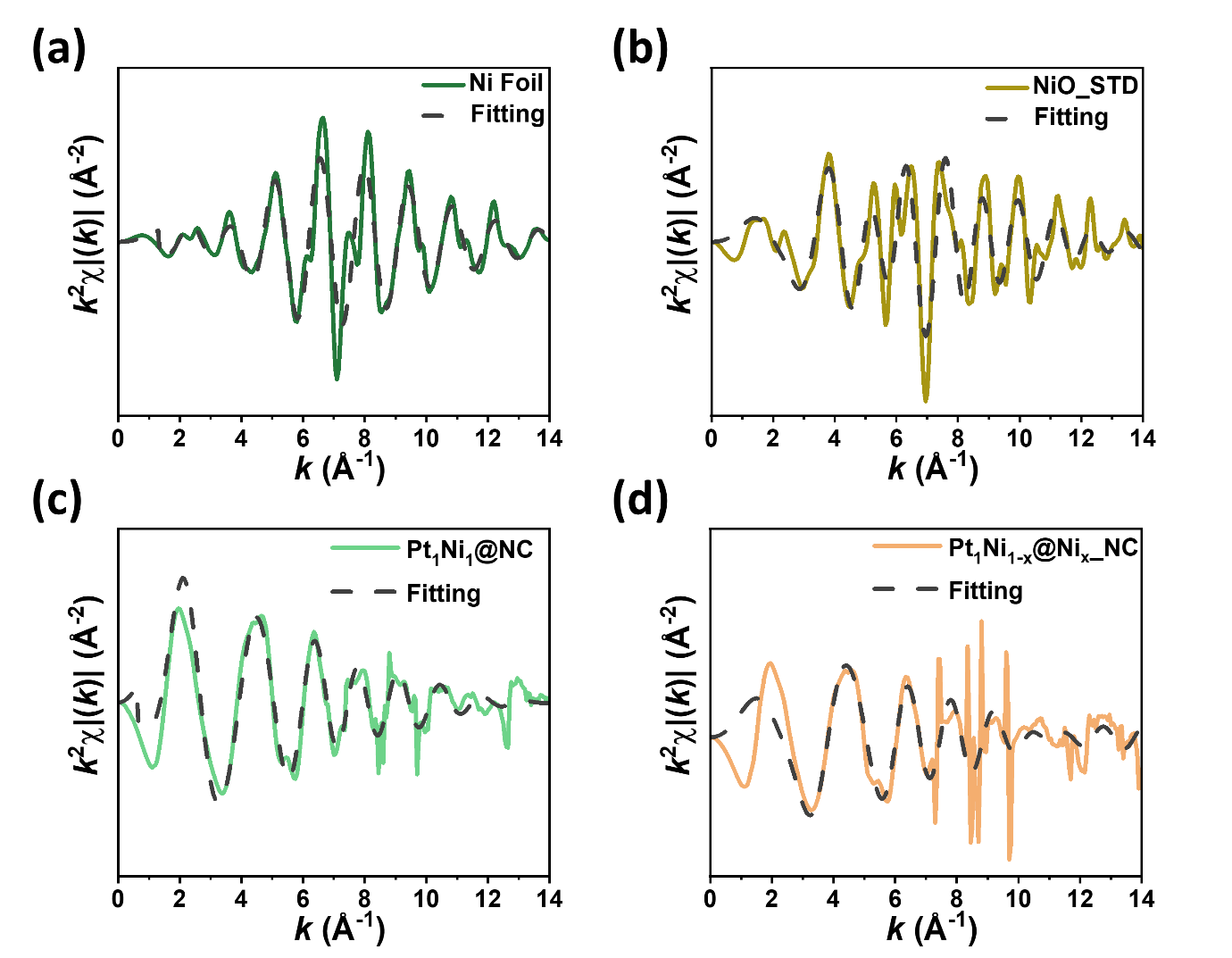
**

**Figure S12.** The experimental data (lines) and fitting data (dots) of Ni K-edge EXAFS spectra for Ni Foil (a), NiO_STD (b), Pt_1_Ni_1_@NC (c) and Pt_1_Ni_1-x_@Ni_x__NC (d), shown in k^2^-weighted K-space.

**
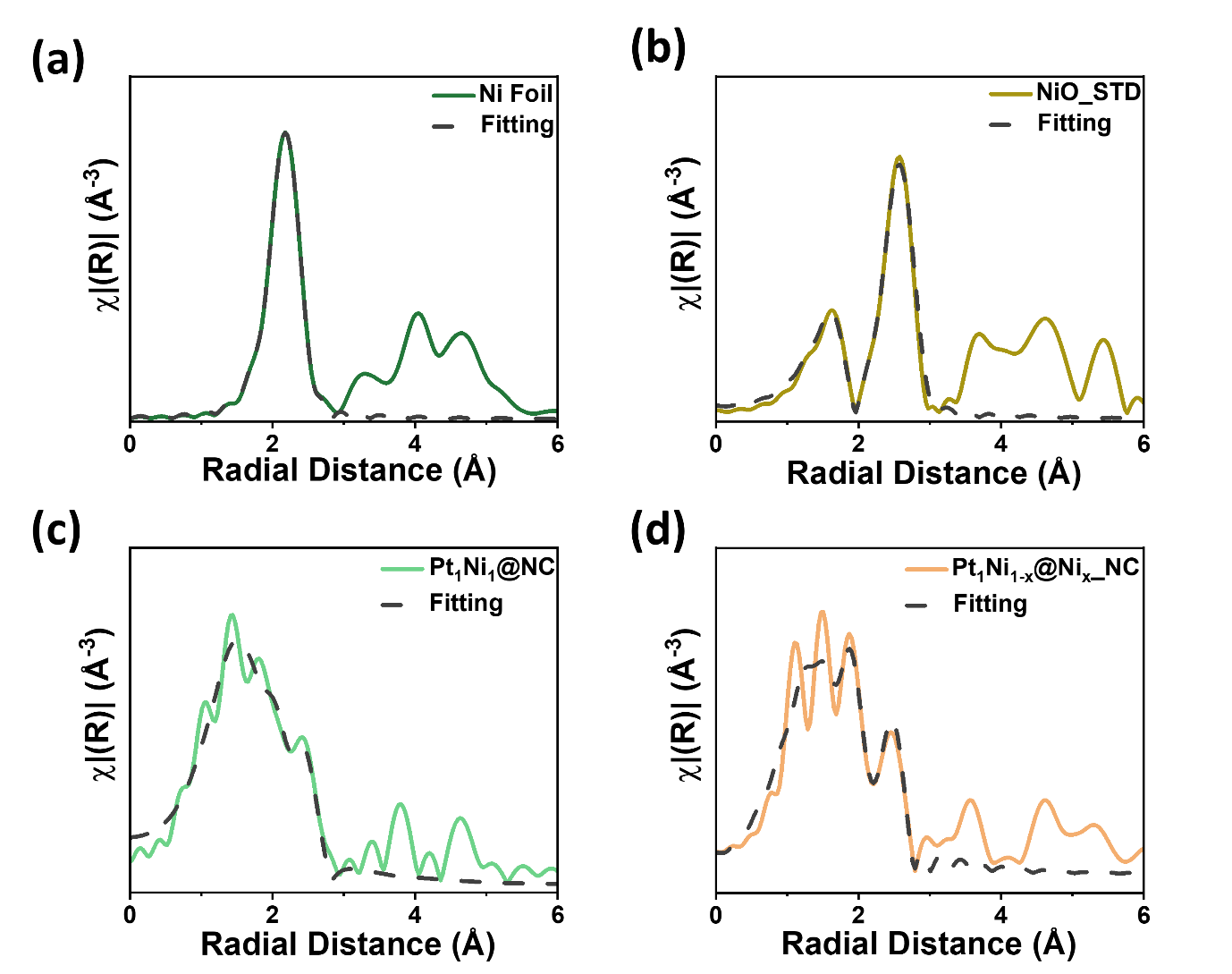
**

**Figure S13.** The experimental data (lines) and fitting data (dots) of Ni K-edge EXAFS spectra for Ni Foil (a), NiO_STD (b), Pt_1_Ni_1_@NC (c) and Pt_1_Ni_1-x_@Ni_x__NC (d), shown in k^2^-weighted R-space.

**
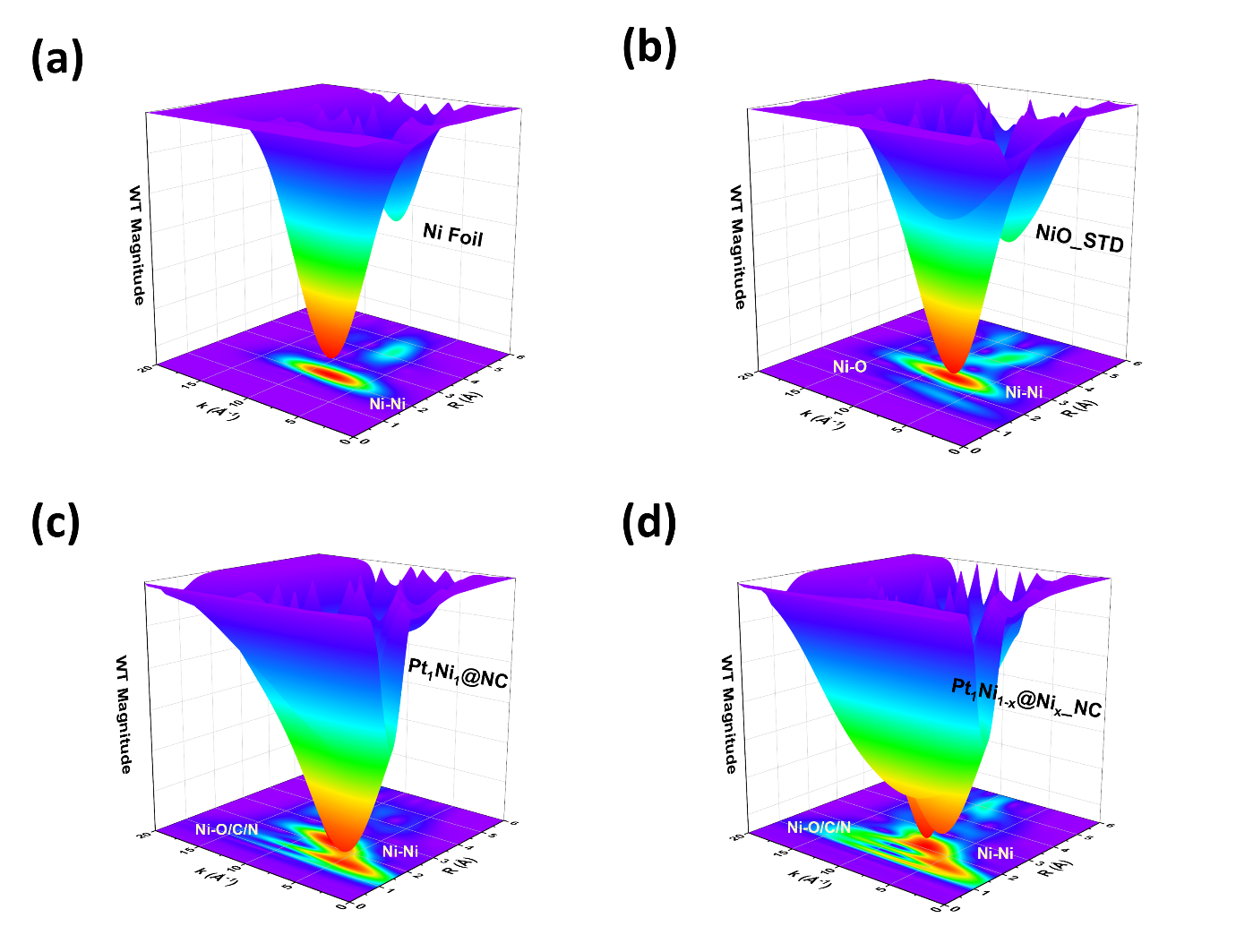
**

**Figure S14.** WT-EXAFS contour maps of the Ni K-edge for Ni Foil (a), NiO_STD (b), Pt_1_Ni_1_@NC (c) and Pt_1_Ni_1-x_@Ni_x__NC (d).

**
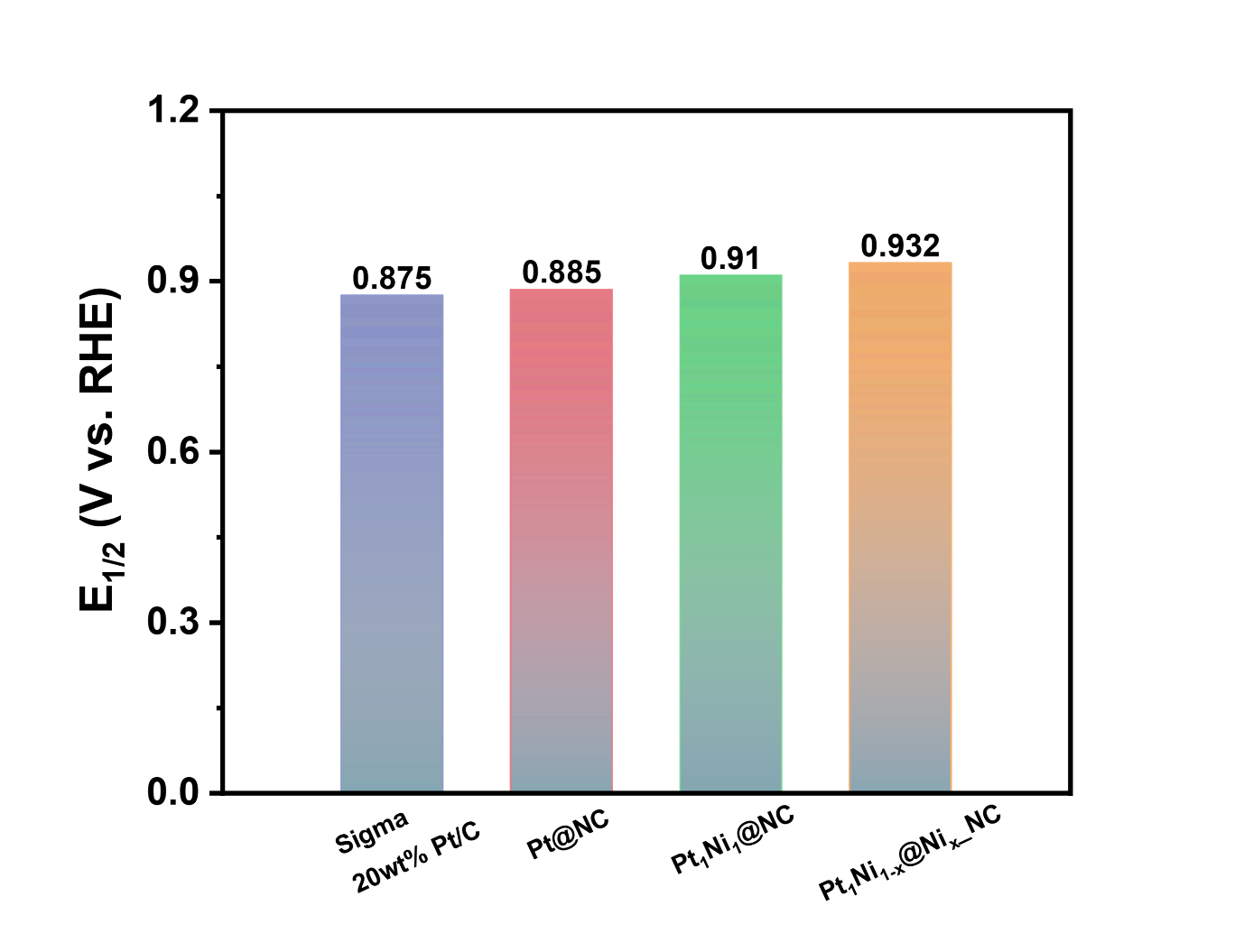
Figure S15.** Half-wave overpotential (E_1/2_) of Sigma 20wt% Pt/C, Pt@NC, Pt_1_Ni_1_@NC and Pt_1_Ni_1-x_@Ni_x__NC.

**
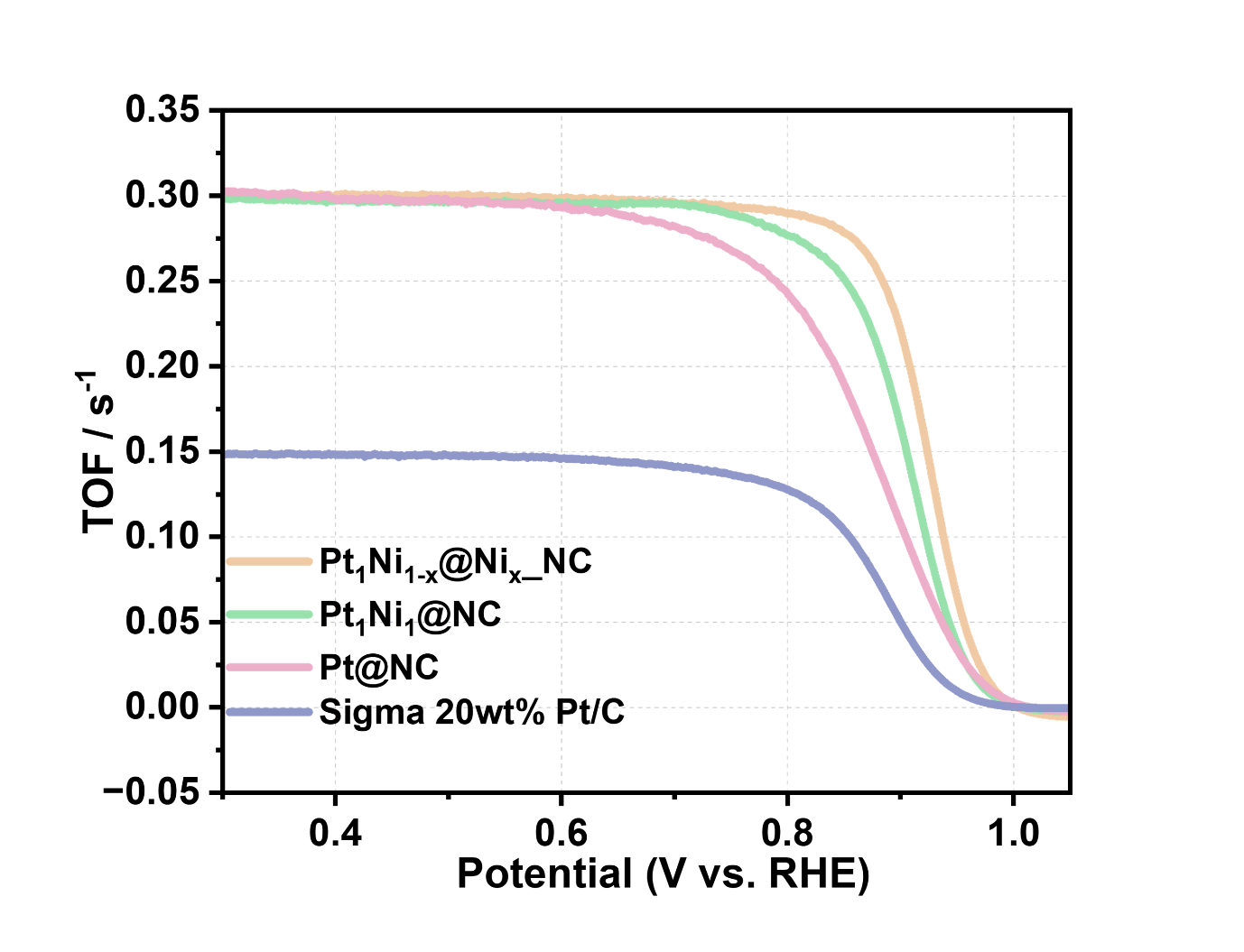
**

**Figure S16.** TOF curves of Sigma 20wt% Pt/C, Pt@NC, Pt_1_Ni_1_@NC and Pt_1_Ni_1-x_@Ni_x__NC.

**
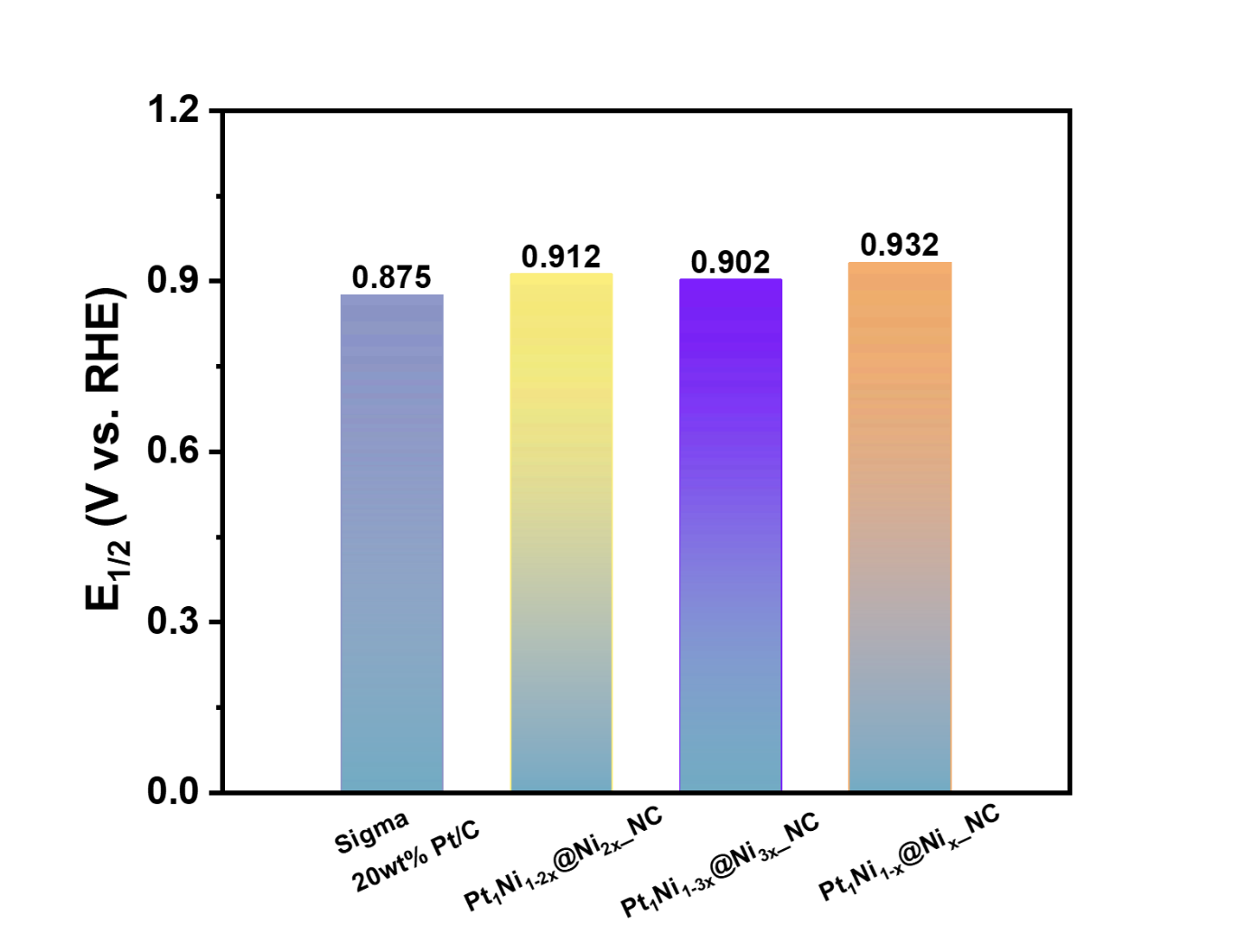
**

**Figure S17.** Half-wave overpotential (E_1/2_) of Sigma 20wt% Pt/C, Pt_1_Ni_1-x_@Ni_x__NC, Pt_1_Ni_1-2x_@Ni_2x__NC and Pt_1_Ni_1-3x_@Ni_3x__NC.


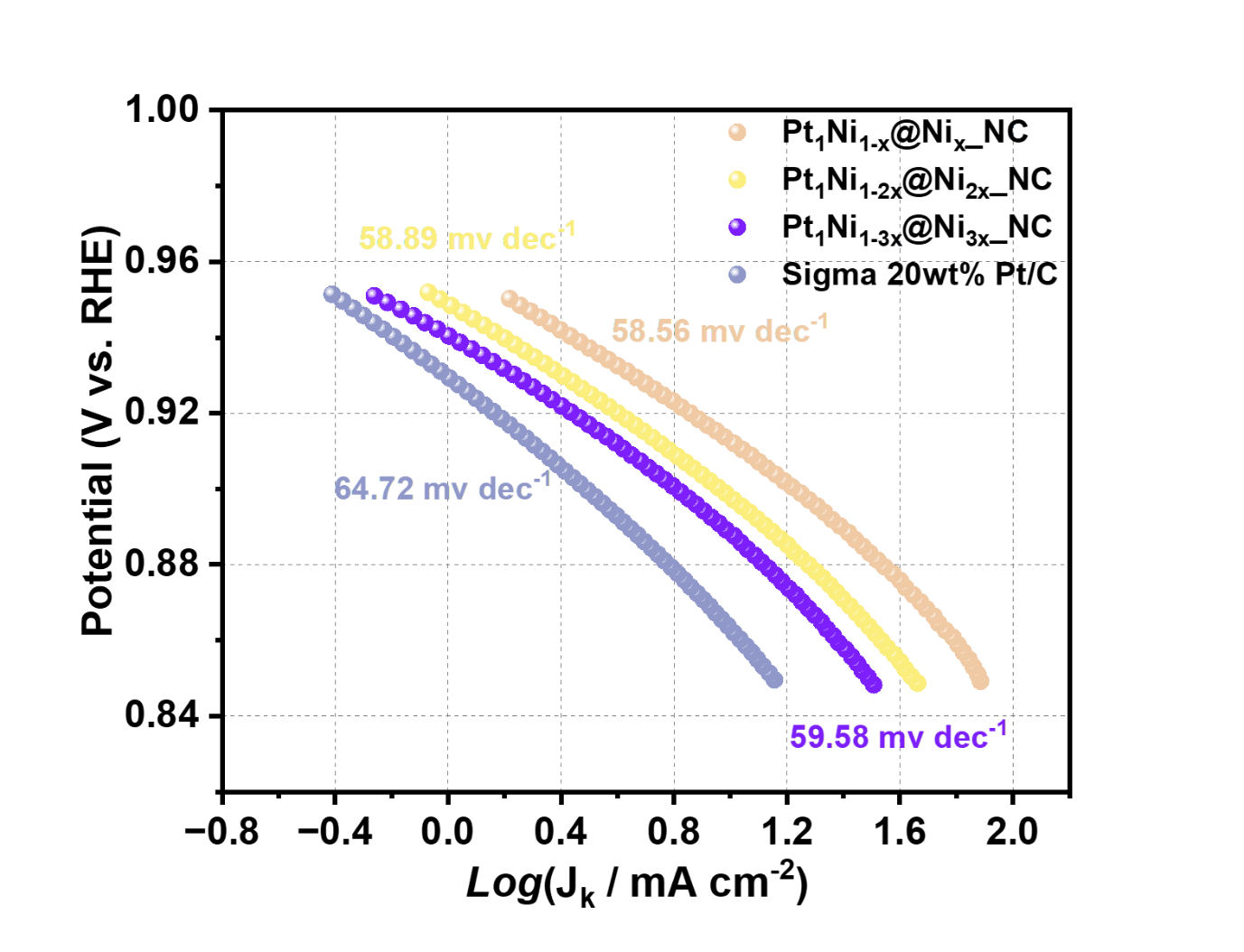


**Figure S18.** Tafel slope of Sigma 20wt% Pt/C, Pt_1_Ni_1-x_@Ni_x__NC, Pt_1_Ni_1-2x_@Ni_2x__NC and Pt_1_Ni_1-3x_@Ni_3x__NC.

**
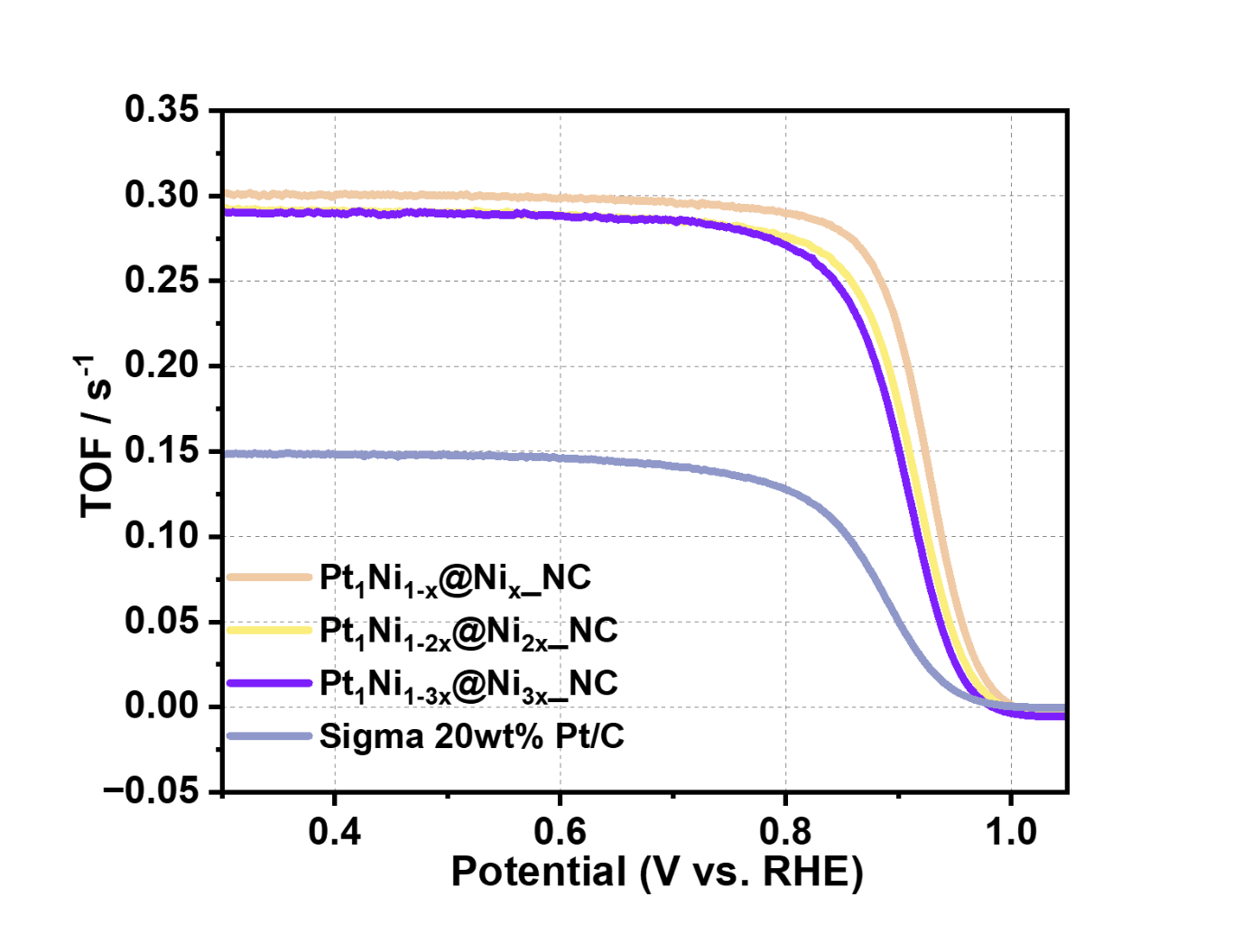
**

**Figure S19.** TOF curves of Sigma 20wt% Pt/C, Pt_1_Ni_1-x_@Ni_x__NC, Pt_1_Ni_1-2x_@Ni_2x__NC and Pt_1_Ni_1-3x_@Ni_3x__NC.

**
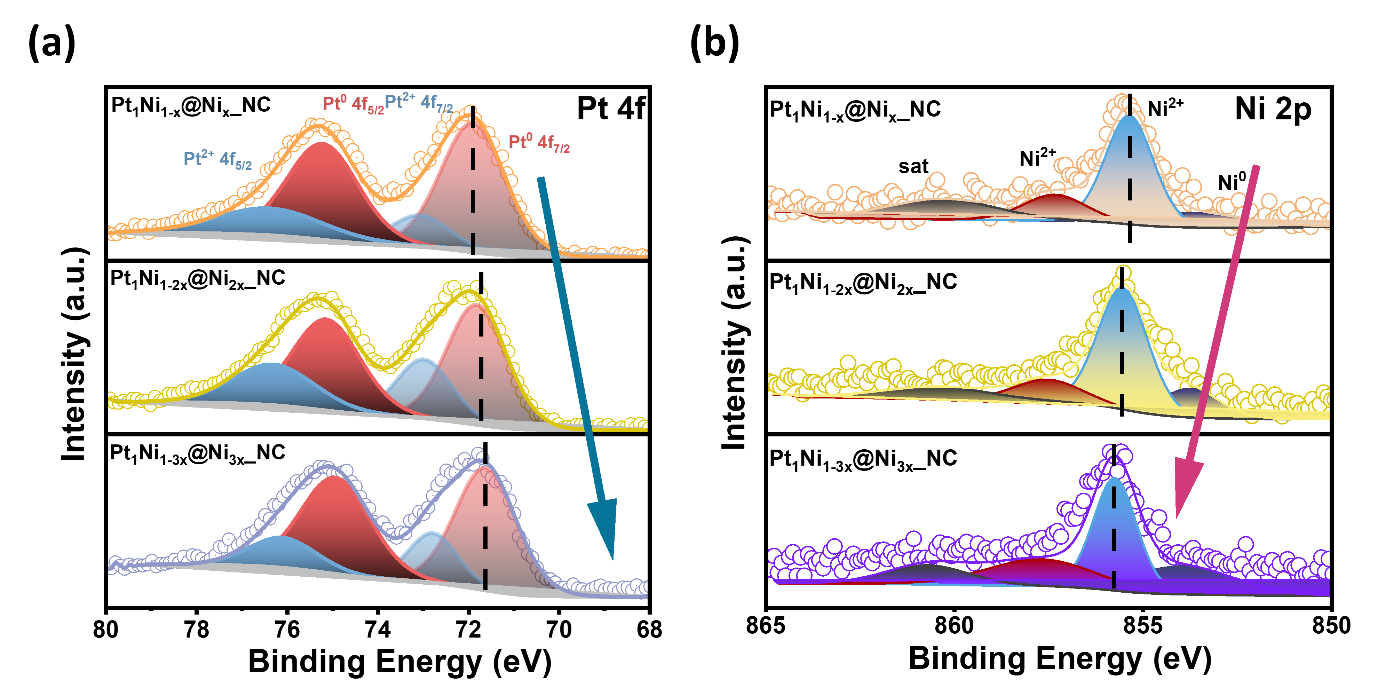
**

**Figure S20.** XPS of Pt_1_Ni_1-x_@Ni_x__NC, Pt_1_Ni_1-2x_@Ni_2x__NC and Pt_1_Ni_1-3x_@Ni_3x__NC, from Pt 4f (a) and Ni 2p (b).

The influence of dealloying temperature on the shell thickness and Pt/Ni compositional gradient was systematically examined by treating the samples at 100, 200, and 300 °C under identical NH₃ flow rates and treatment durations. Increasing the dealloying temperature progressively promoted near-surface Ni extraction, resulting in a more pronounced Pt/Ni gradient, whereas excessively high temperatures induced partial structural relaxation. Ni K-edge XANES analysis shows that the white-line intensity initially increases and subsequently decreases with increasing temperature, suggesting enhanced Ni dissolution followed by re-aggregation at higher temperatures. Consistently, the sample treated at 300 °C exhibits a reduced Ni white-line intensity and the lowest Pt–Ni coordination number, indicating extensive Ni depletion from the alloyed framework. (**Figure 4f**, **Figure S21-23**, **Table S4-5**, **8-9**)

**
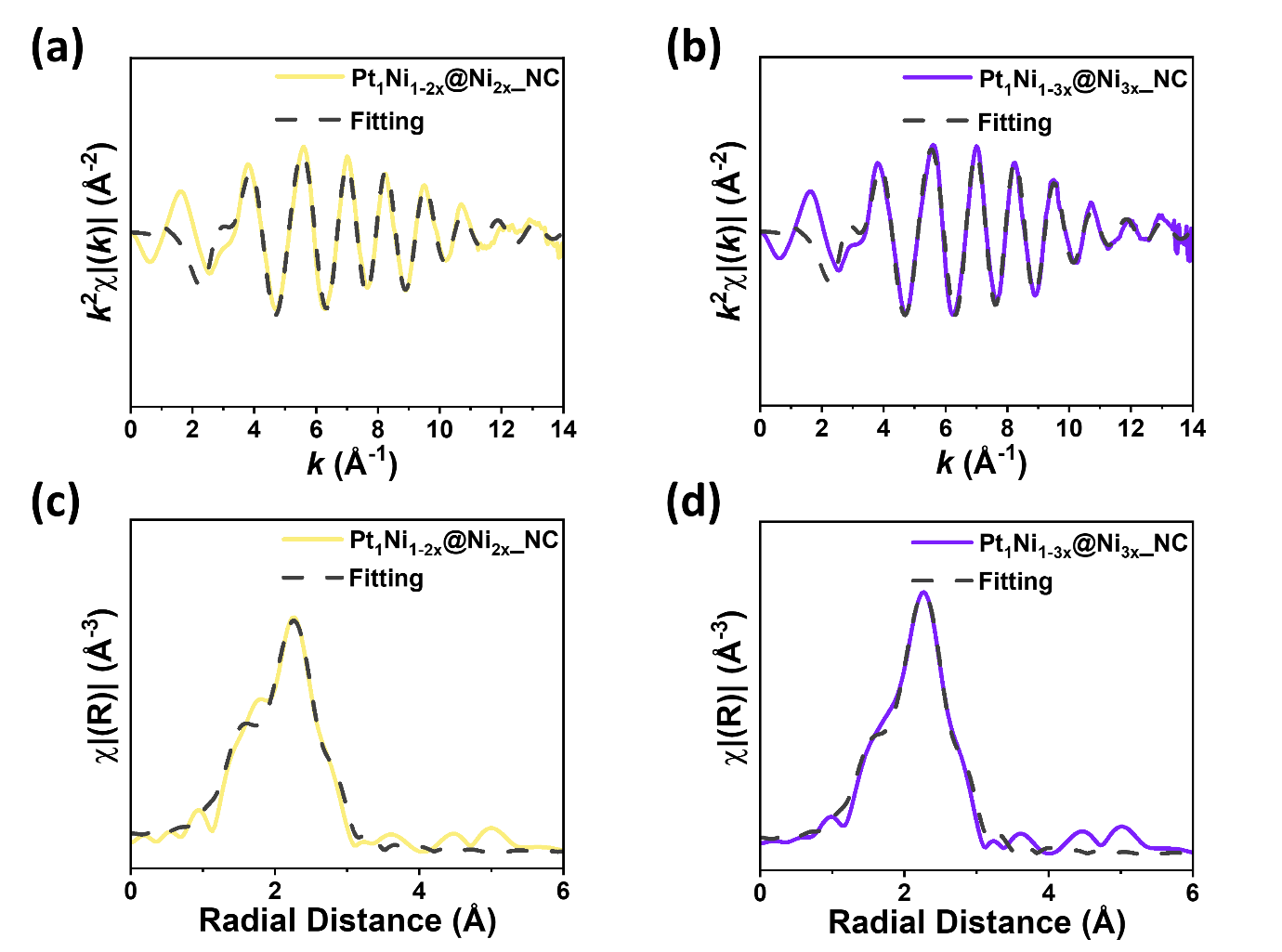
**

**Figure S21.** The experimental data (lines) and fitting data (dots) of Pt L_3_-edge EXAFS spectra for Pt_1_Ni_1-2x_@Ni_2x__NC (a, c) and Pt_1_Ni_1-3x_@Ni_3x__NC (b, d), shown in k^2^-weighted K-space and R-space.

**
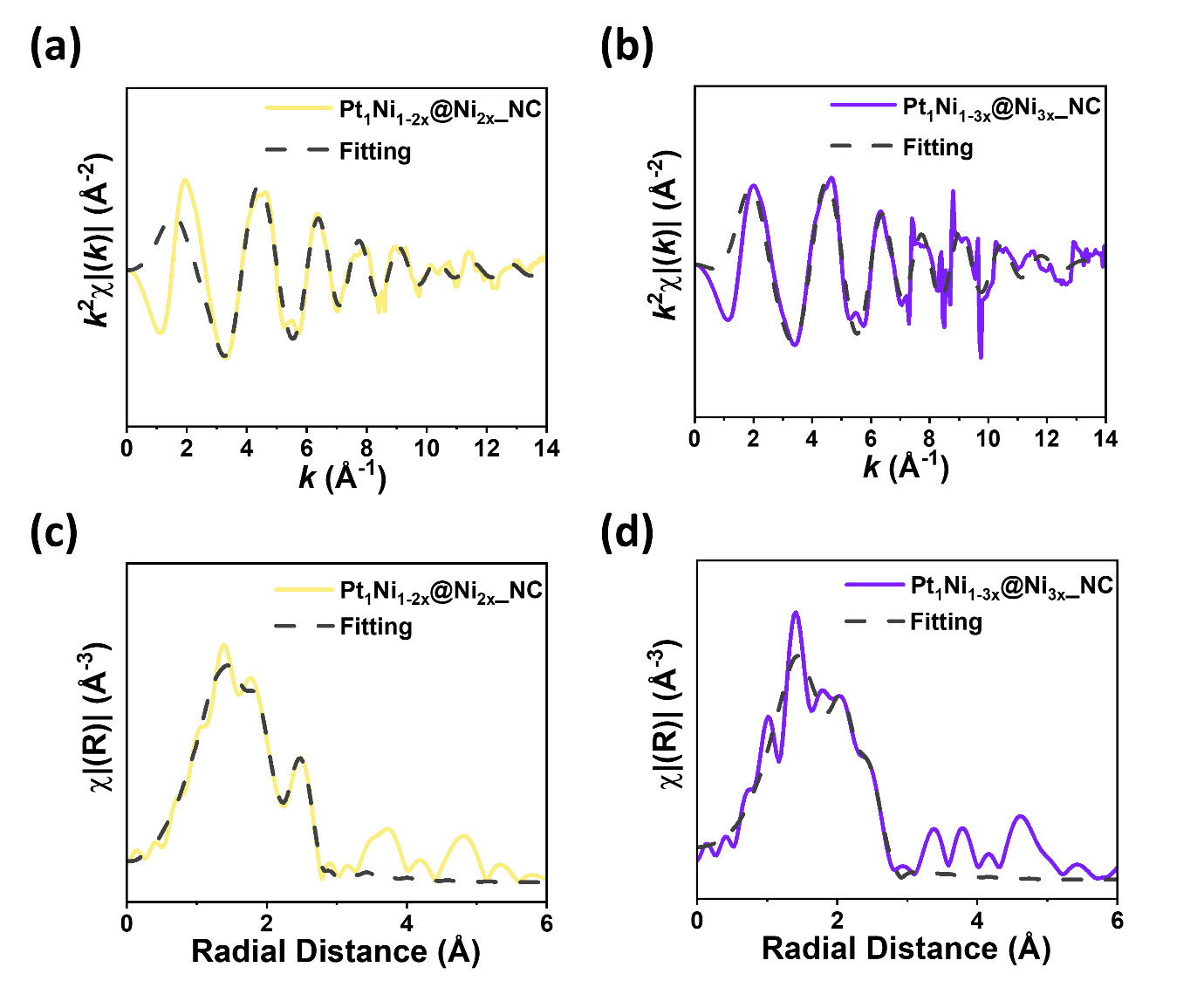
**

**Figure S22.** The experimental data (lines) and fitting data (dots) of Ni K-edge EXAFS spectra for Pt_1_Ni_1-2x_@Ni_2x__NC (a, c) and Pt_1_Ni_1-3x_@Ni_3x__NC (b, d), shown in k^2^-weighted K-space and R-space.

**
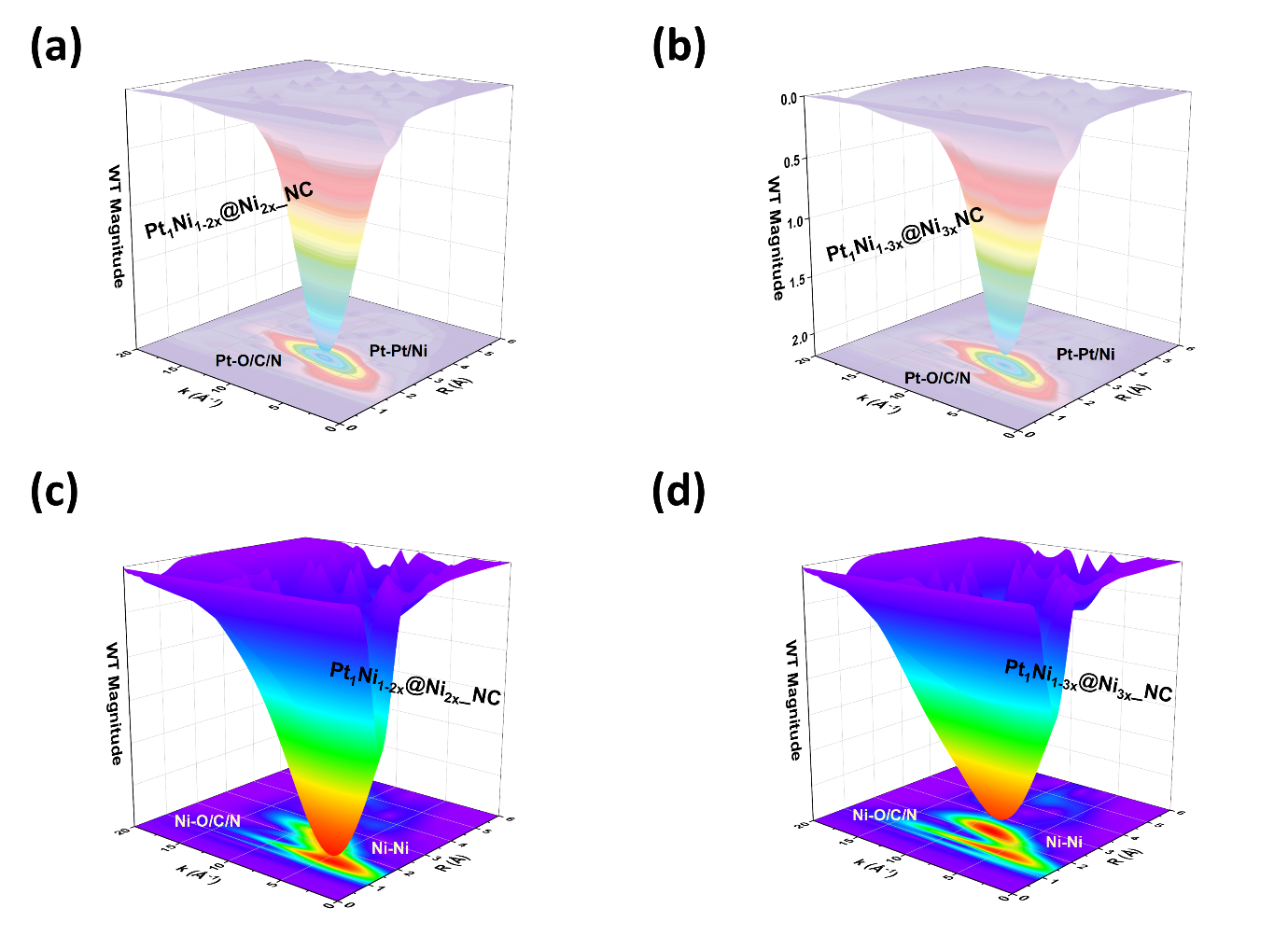
**

**Figure S23.** WT-EXAFS contour maps of the Pt_1_Ni_1-2x_@Ni_2x__NC (a, c) and Pt_1_Ni_1-3x_@Ni_3x__NC (b, d) from Pt-L_3_ edge and Ni-K edge.

**
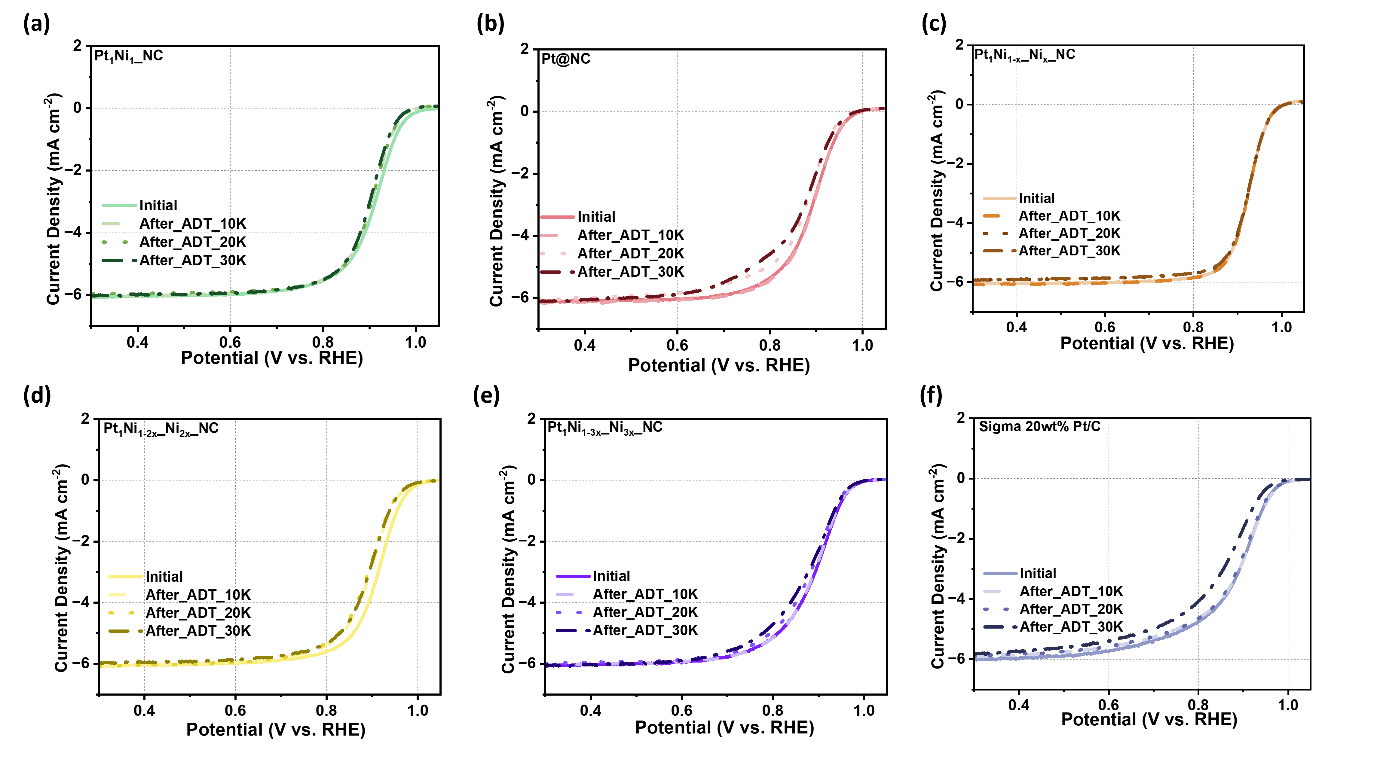
**

**Figure S24.** ORR polarization curves before and after 10/20/30k ADT of Pt_1_Ni_1_@NC (a), Pt@NC (b), Pt_1_Ni_1-x_@Ni_x__NC (c), Pt_1_Ni_1-2x_@Ni_2x__NC (d), Pt_1_Ni_1-3x_@Ni_3x__NC (e) and Sigma 20wt% Pt/C (f).

**
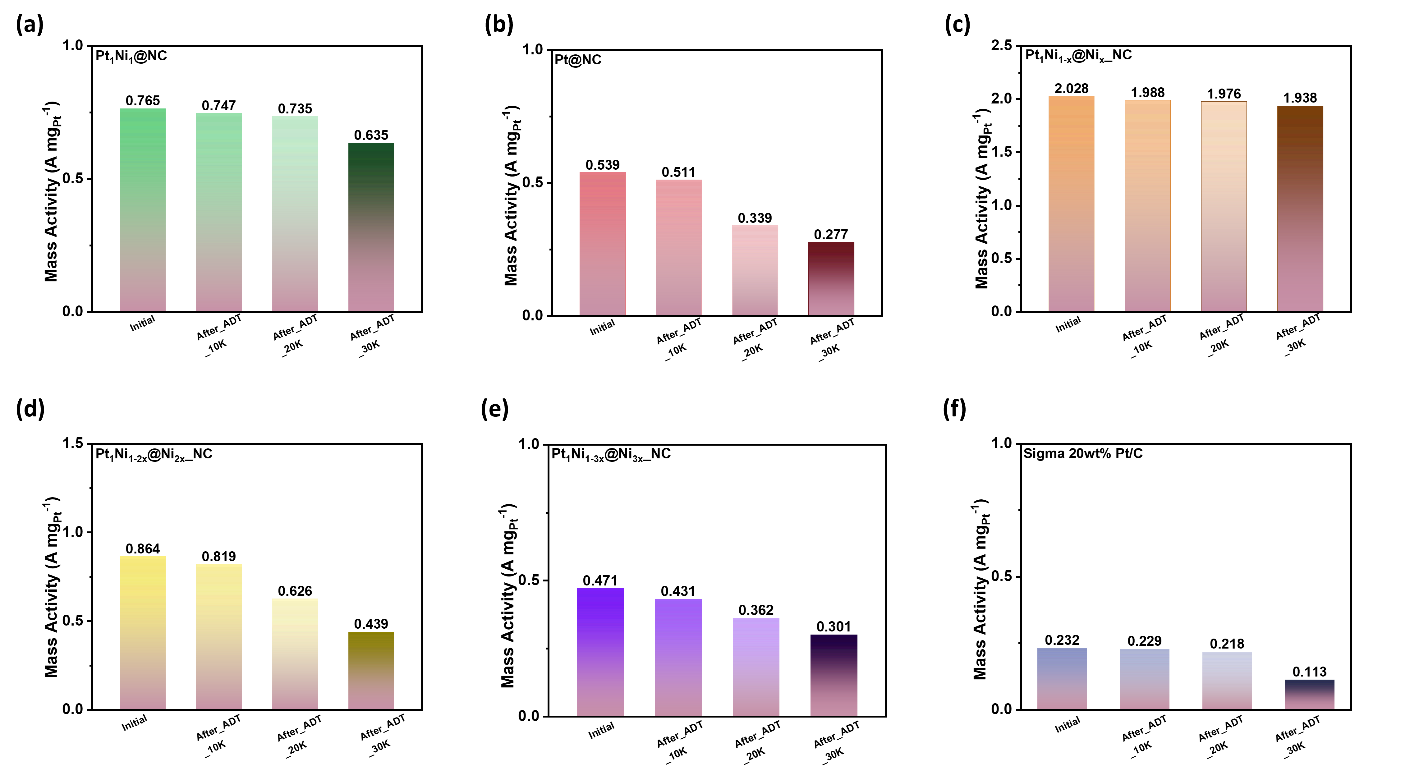
**

**Figure S25.** Mass activity at 0.9 V *vs.*RHE of Pt_1_Ni_1_@NC (a), Pt@NC (b), Pt_1_Ni_1-x_@Ni_x__NC (c), Pt_1_Ni_1-2x_@Ni_2x__NC (d), Pt_1_Ni_1-3x_@Ni_3x__NC (e) and Sigma 20wt% Pt/C (f).

**
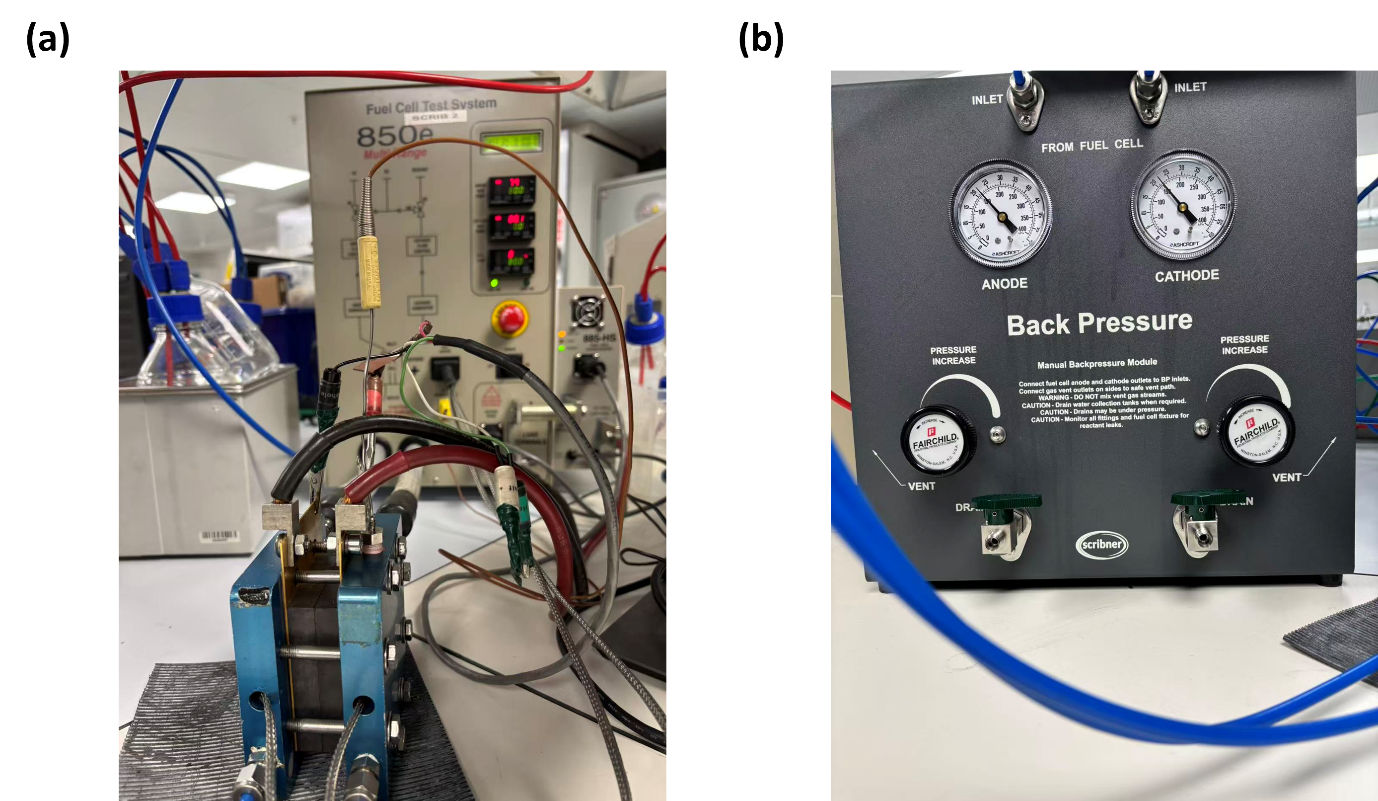
**

**Figure S26.** (a) 850e Multi-Range Fuel Cell Test System (Scribner, LLC.) and MEA Stack. (b) Backpressure device.

**
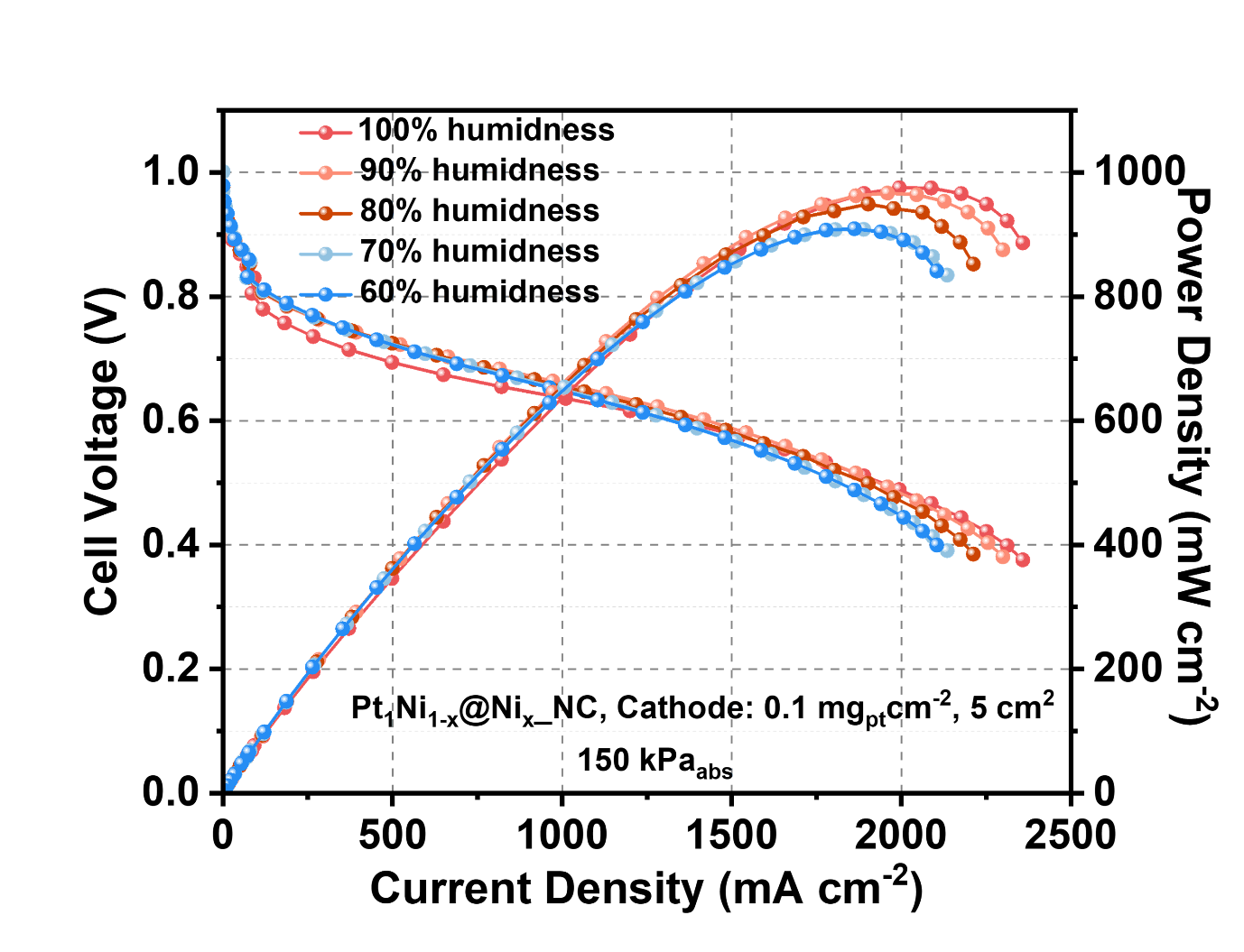
**

**Figure S27.** Humidness dependence pf H_2_-air MEA performance of Pt_1_Ni_1-x_@Ni_x__NC under practical operating conditions.

**
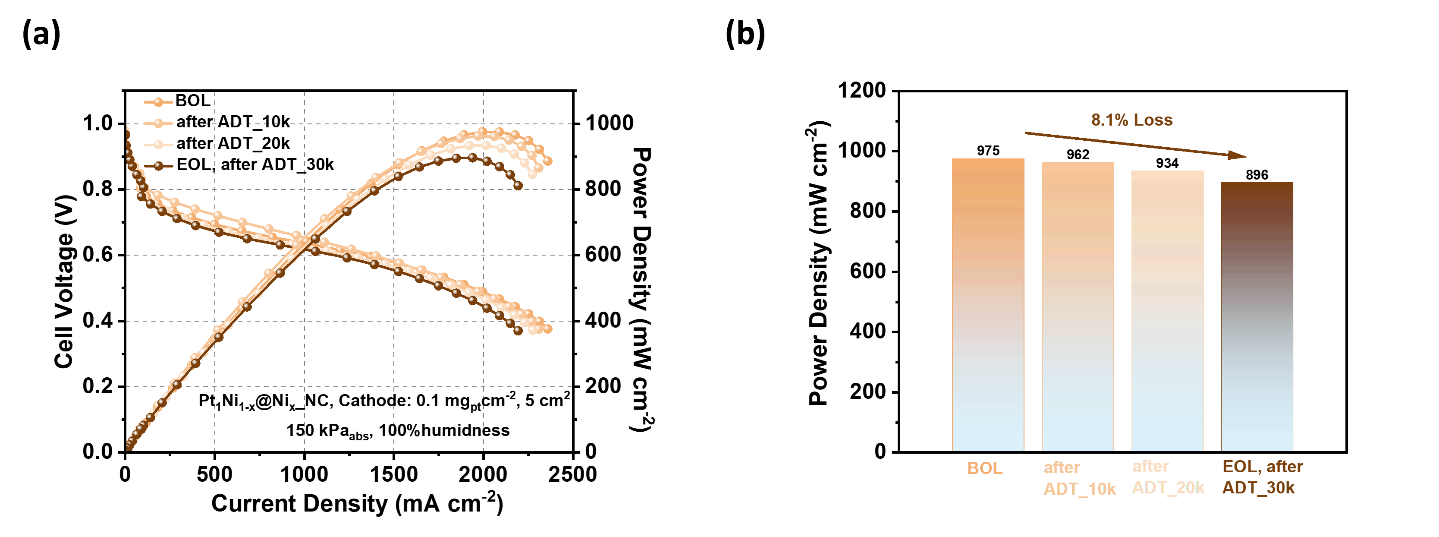
**

**Figure S28.** (a) H_2_-air MEA performance before (BOL) and after ADT (10-30k cycles, EOL) of Pt_1_Ni_1-x_@Ni_x__NC under practical operating conditions. (b) Peak power density before and after ADT.

**
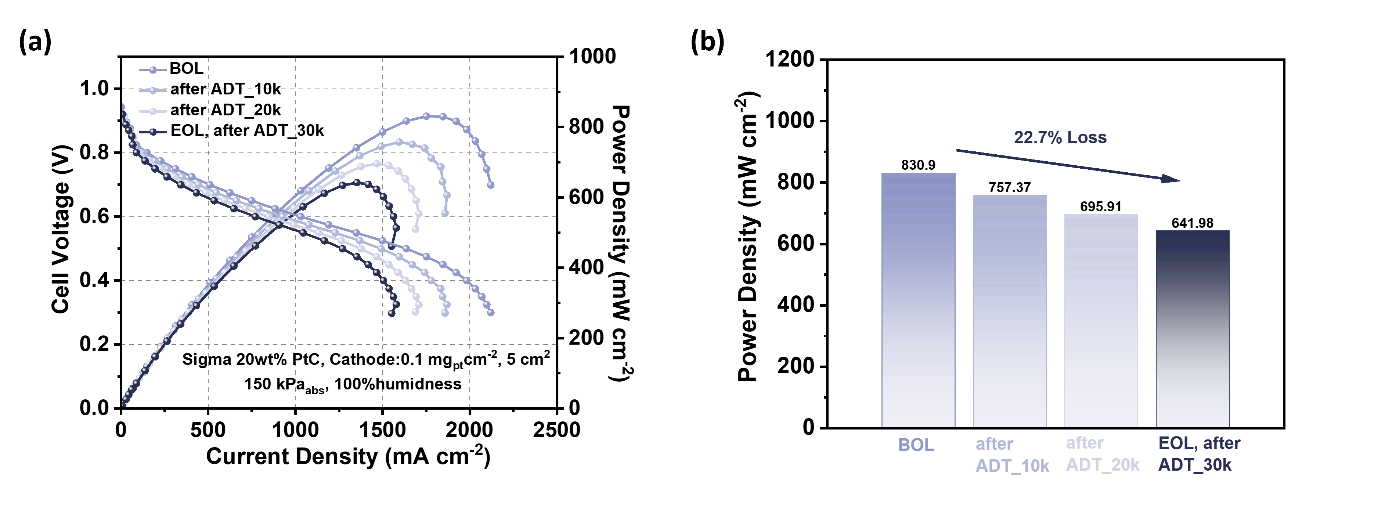
**

**Figure S29.** (a) H_2_-air MEA performance before (BOL) and after ADT (10-30k cycles, EOL) of Sigma 20wt% Pt/C under practical operating conditions. (b) Peak power density before and after ADT.

**
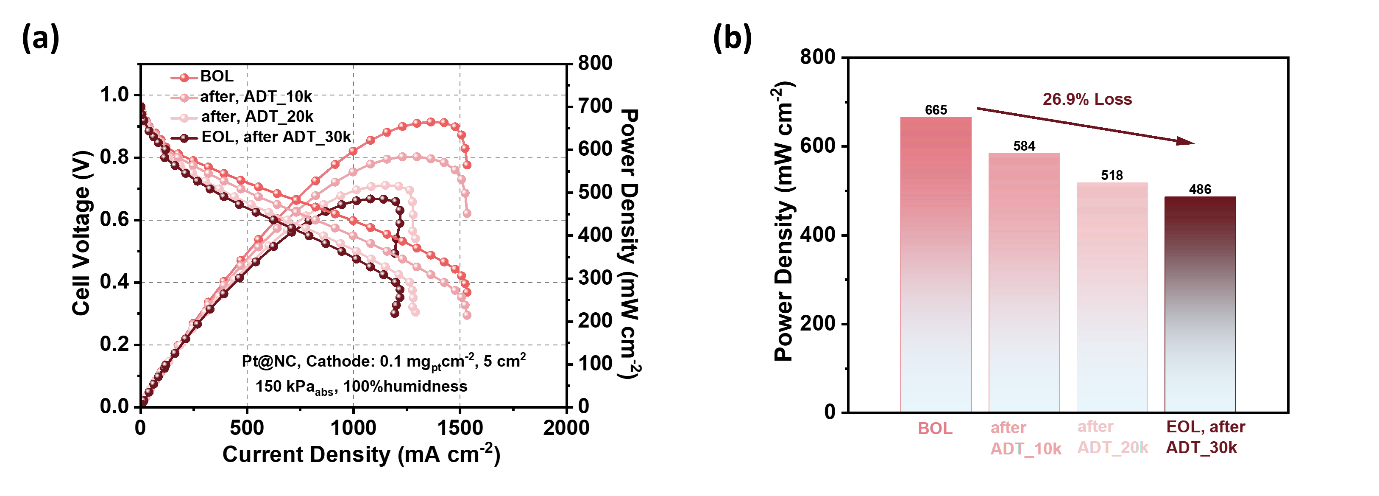
**

**Figure S30.** (a) H_2_-air MEA performance before (BOL) and after ADT (10-30k cycles, EOL) of Pt@NC under practical operating conditions. (b) Peak power density before and after ADT.

**
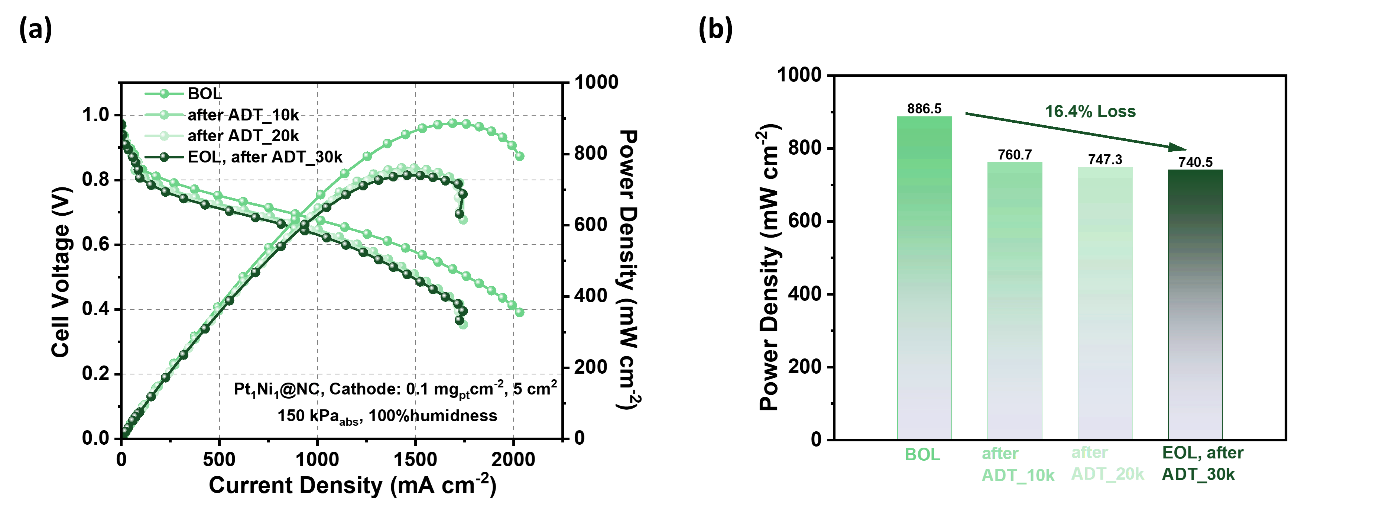
**

**Figure S31.** (a) H_2_-air MEA performance before (BOL) and after ADT (10-30k cycles, EOL) of Pt_1_Ni_1_@NC under practical operating conditions. (b) Peak power density before and after ADT.

**Table S1.** The Gibbs free energy of corresponding reaction coordinate of ORR process on Pt, Pt_3_Ni and Pt@Pt_3_Ni.

| **Gibbs free energy (eV)**  **Sample** | ***OOH** | ***O** | ***OH** |
| --- | --- | --- | --- |
| **Pt** | 0.06816 | -1.26796 | -0.32166 |
| **Pt_3_Ni** | 0.30161 | -0.98647 | -0.20897 |
| **Pt@Pt_3_Ni** | 0.21682 | -0.63383 | -0.10041 |

**Table S2.** Pt 4f XPS spectra of prepared catalysts.

| **Sample name** | **Pt^0^4f_7/2_ (eV)** | **Pt^2+^4f_7/2_ (eV)** | **Pt^0^4f_5/2_ (eV)** | **Pt^2+^4f_5/2_ (eV)** |
| --- | --- | --- | --- | --- |
| **Pt@NC** | 70.65 | 71.81 | 73.97 | 75.13 |
| **Pt_1_Ni_1_@ NC** | 71.64 | 72.80 | 74.96 | 76.12 |
| **Pt_1_Ni_1-x_@Ni_x__NC** | 72.10 | 73.26 | 75.42 | 76.58 |

**Table S3.** Ni 2p_3/2_ XPS spectra of prepared catalysts.

| **Sample name** | **Ni^0^2p_3/2_ (eV)** | **Ni^2+^2p_3/2_ (eV)** | **Ni^2+^4p_3/2_ (eV)** |
| --- | --- | --- | --- |
| **Ni@NC** | 852.60 | 854.41 | 856.38 |
| **Pt_1_Ni_1_@ NC** | 853.67 | 855.48 | 857.45 |
| **Pt_1_Ni_1-x_@Ni_x__NC** | 853.78 | 855.59 | 857.56 |

**Table S4.** Pt-L_3_ edge EXAFS fitting results of Pt Foil, Pt_1_Ni_1_@NC, and Pt_1_Ni_1-x_@Ni_x__NC.

| **EXAFS scattering paths** | **Parameters** | **Pt Foil** | **Pt_1_Ni_1_@NC** | **Pt_1_Ni_1-x_@Ni_x__NC** |
| --- | --- | --- | --- | --- |
| **Pt-Pt**  **(Pt L_3_-edge)** | **C.N.** | 12 ± 0.5 | 6.4 ± 1.4 | 5.3 ± 1.5 |
|  | **R [Å]** | 2.72 ± 0.005 | 2.72 ± 0.01 | 2.72 ± 0.02 |
|  | **σ^2^** | 0.004 ± 0.0002 | 0.01 ± 0.001 | 0.01 ± 0.001 |
| **Pt-Ni**  **(Pt L_3_-edge)** | **C.N.** | N.A. | 2.2 ± 0.8 | 2.1 ± 0.9 |
|  | **R [Å]** | N.A. | 2.64 ± 0.08 | 2.63 ± 0.04 |
|  | **σ^2^** | N.A. | 0.009 ± 0.003 | 0.009 ± 0.003 |
| **Pt-C/N/O**  **(Pt L_3_-edge)** | **C.N.** | N.A. | 2.2 ± 0.5 | 3.0 ± 0.5 |
|  | **R [Å]** | N.A. | 1.99 ± 0.03 | 1.97 ± 0.02 |
|  | **σ^2^** | N.A. | 0.009 ± 0.005 | 0.009 ± 0.005 |

**Table S5.** Ni-K edge EXAFS fitting results of Ni Foil, NiO_STD, Pt_1_Ni_1__NC and Pt_1_Ni_1-x__Ni_x__NC.

| **EXAFS scattering paths** | **Parameters** | **Ni Foil** | **NiO_STD** | **Pt_1_Ni_1_@NC** | **Pt_1_Ni_1-x_@Ni_x_NC** |
| --- | --- | --- | --- | --- | --- |
| **Ni-Ni**  **(Ni K-edge)** | **C.N.** | 12 ± 0.3 | 10.7 ± 0.5 | 1.0 ± 0.6 | 0.5 ± 0.8 |
|  | **R [Å]** | 2.49 ± 0.02 | 2.54 ± 0.01 | 2.53 ± 0.03 | 2.41 ± 0.08 |
|  | **σ^2^** | 0.006 ± 0.0002 | 0.007 ± 0.0007 | 0.006 ± 0.004 | 0.003 ± 0.007 |
| **Ni-Pt**  **(Ni K-edge)** | **C.N.** | N.A. | N.A. | 1.1 ± 1.3 | 2.6 ± 1.2 |
|  | **R [Å]** | N.A. | N.A. | 2.62 ± 0.04 | 2.55 ± 0.06 |
|  | **σ^2^** | N.A. | N.A. | 0.009 ± 0.008 | 0.008 ± 0.007 |
| **Ni-C/N/O**  **(Ni K-edge)** | **C.N.** | N.A. | 5.3 ± 0.7 | 9.6 ± 1.8 | 10.2 ± 4.5 |
|  | **R [Å]** | N.A. | 2.08 ± 0.06 | 1.95 ± 0.05 | 1.84 ± 0.11 |
|  | **σ^2^** | N.A. | 0.006 ± 0.009 | 0.02 ± 0.008 | 0.02 ± 0.16 |

**Table S6.** Pt 4f XPS spectra of prepared catalysts.

| **Sample name** | **Pt^0^4f_7/2_ (eV)** | **Pt^2+^4f_7/2_ (eV)** | | **Pt^0^4f_5/2_ (eV)** | **Pt^2+^4f_5/2_ (eV)** |
| --- | --- | --- | --- | --- | --- |
| **Pt_1_Ni_1-x_@Ni_x__NC** | 72.10 | 73.26 | 75.42 | | 76.58 |
| **Pt_1_Ni_1-2x_@Ni_2x__NC** | 71.98 | 73.14 | 75.30 | | 76.46 |
| **Pt_1_Ni_1-3x_@Ni_3x__NC** | 71.71 | 72.87 | 75.03 | | 76.19 |

**Table S7.** Ni 2p_3/2_ XPS spectra of prepared catalysts.

| **Sample name** | **Ni^0^2p_3/2_ (eV)** | **Ni^2+^2p_3/2_ (eV)** | **Ni^2+^4p_3/2_ (eV)** |
| --- | --- | --- | --- |
| **Pt_1_Ni_1-x_@Ni_x__NC** | 853.78 | 855.59 | 857.56 |
| **Pt_1_Ni_1-2x_@Ni_2x__NC** | 853.90 | 855.71 | 857.68 |
| **Pt_1_Ni_1-3x_@Ni_3x__NC** | 854.24 | 856.05 | 858.02 |

**Table S8.** Pt-L_3_ edge EXAFS fitting results of Pt_1_Ni_1-2x_@Ni_2x__NC and Pt_1_Ni_1-3x_@Ni_3x__NC.

| **EXAFS scattering paths** | **Parameters** | **Pt_1_Ni_1-2x_@Ni_2x__NC** | **Pt_1_Ni_1-3x_@Ni_3x__NC** |
| --- | --- | --- | --- |
| **Pt-Pt**  **(Pt L_3_-edge)** | **C.N.** | 5.4 ± 1.4 | 5.6 ± 1.4 |
|  | **R [Å]** | 2.72 ± 0.02 | 2.72 ± 0.02 |
|  | **σ^2^** | 0.01 ± 0.001 | 0.01 ± 0.001 |
| **Pt-Ni**  **(Pt L_3_-edge)** | **C.N.** | 1.9 ± 0.8 | 1.6 ± 0.5 |
|  | **R [Å]** | 2.63 ± 0.02 | 2.64 ± 0.02 |
|  | **σ^2^** | 0.009 ± 0.003 | 0.009 ± 0.003 |
| **Pt-C/N/O**  **(Pt L_3_-edge)** | **C.N.** | 3.0 ± 0.5 | 3.1 ± 0.3 |
|  | **R [Å]** | 1.97 ± 0.02 | 1.97 ± 0.03 |
|  | **σ^2^** | 0.009 ± 0.005 | 0.009 ± 0.005 |

**Table S9.** Ni-K edge EXAFS fitting results of Pt_1_Ni_1-2x_@Ni_2x__NC and Pt_1_Ni_1-3x_@Ni_3x__NC.

| **EXAFS scattering paths** | **Parameters** | **Pt_1_Ni_1-2x_@Ni_2x__NC** | **Pt_1_Ni_1-3x_@Ni_3x__NC** |
| --- | --- | --- | --- |
| **Ni-Ni**  **(Ni K-edge)** | **C.N.** | 1.1 ± 0.7 | 1.5± 0.5 |
|  | **R [Å]** | 2.46 ± 0.04 | 2.46 ± 0.01 |
|  | **σ^2^** | 0.01 ± 0.007 | 0.009 ± 0.0007 |
| **Ni-Pt**  **(Ni K-edge)** | **C.N.** | 2.4 ± 1.5 | 2.2 ± 1.5 |
|  | **R [Å]** | 2.57 ± 0.03 | 2.57 ± 0.03 |
|  | **σ^2^** | 0.009 ± 0.003 | 0.009 ± 0.003 |
| **Ni-C/N/O**  **(Ni K-edge)** | **C.N.** | 10.0 ± 1.5 | 9.8 ± 1.5 |
|  | **R [Å]** | 1.86 ± 0.05 | 1.91 ± 0.05 |
|  | **σ^2^** | 0.02 ± 0.006 | 0.02 ± 0.006 |

**Table S10.** ORR performance comparison between electrocatalysts in this work and other recent reported Pt-based electrocatalysts.

| **Catalyst** | **Half wave overpotential (V)** | **Tafel slope (mV/dec)** | **Mass activity**  **@0.9 V (A mg_pt_^-1^)** | **Ref.** |
| --- | --- | --- | --- | --- |
| **L1_0_-Cr-PtFe/C** | **0.952** | **N.A.** | **2.05** | **1^[5]^** |
| **PtFe/NcPS** | **~0.91** | **N.A.** | **1.35** | **2^[6]^** |
| **PtCo/Sn–N–C** | **~0.91** | **N.A.** | **1.65** | **3^[7]^** |
| **L1_2_-Pt_3_Co/Ti-a-NPC** | **0.936** | **N.A.** | **1.765** | **4^[8]^** |
| **700-Pt1Co1-IMC@Pt/C-2.5** | **~0.91** | **N.A.** | **0.53** | **5^[9]^** |
| **PtCo/P_2.73_O*_x_*–KB** | **0.928** | **N.A.** | **1.11** | **6^[10]^** |
| **HEI@Pt/C** | **~0.90** | **62.2** | **0.65** | **7^[11]^** |
| **PNAC-*o*-Pt_3_Fe/C** | **0.926** | **53.0** | **1.66** | **8^[12]^** |
| **PtFe@Fe_SAs_-N-C** | **0.872** | **N.A.** | **1.18** | **9^[13]^** |
| **DC-Pt_1_Co_1_/CNT** | **0.94** | **N.A.** | **2.16** | **10^[14]^** |
| **Pt_3_Ga*(Pm*3¯*m)*** | **0.941** | **61.2** | **2.18** | **11^[15]^** |
| **L1_0_-PtCo_0.50_Fe_0.50_** | **0.945** | **69.96** | **0.93** | **12^[16]^** |
| **PtCo@Pt/C-6** | **~0.91** | **N.A.** | **0.996** | **13^[17]^** |
| **Pt@PtCu NWs** | **0.94** | **60** | **1.17** | **14^[18]^** |
| **Pt@m-TiCN NFs** | **0.89** | **N.A.** | **0.3** | **15^[19]^** |
| **Pt_1_Ni_1__NC** | **0.910** | **60.87** | **0.765** | **This work** |
| **Pt@NC** | **0.885** | **79.41** | **0.718** | **This work** |
| **Pt_1_Ni_1-x_@Ni_x__NC** | **0.932** | **58.56** | **2.028** | **This work** |
| **Pt_1_Ni_1-2x_@Ni_2x__NC** | **0.912** | **58.89** | **0.864** | **This work** |
| **Pt_1_Ni_1-3x_@Ni_3x__NC** | **0.902** | **59.58** | **0.471** | **This work** |
| **Sigma 20wt% Pt/C** | **0.875** | **64.72** | **0.232** | **This work** |

**Table S11.** ICP results of prepared catalysts.

| **Sample name** | **Pt (wt.%)** | **Ni (wt.%)** |
| --- | --- | --- |
| **Pt@NC** | 14.65 | N.A. |
| **Pt_1_Ni_1_@ NC** | 11.66 | 3.41 |
| **Pt_1_Ni_1-x_@Ni_x__NC** | 10.23 | 2.88 |
| **Pt_1_Ni_1-2x_@Ni_2x__NC** | 10.41 | 2.45 |
| **Pt_1_Ni_1-3x_@Ni_3x__NC** | 10.18 | 2.15 |
| **Com. 20 wt%Pt/C** | 20 | N.A. |

**Table S12.** PEMFCs performance comparison between electrocatalysts in this work and other recent reported Pt-based electrocatalysts.

| **Catalyst** | **Cathode loading**  **(mg Pt cm^-2^)** | **Back**  **Pressure (abs)** | **Peak Power Density (mW cm^-2^)** | **Loss after ADT (%)** | **Ref.** |
| --- | --- | --- | --- | --- | --- |
| **PtCoAu_1.5_ NWs** | **0.20** | **N.A.** | **740** | **8 (after 10k)** | **1^[20]^** |
| **Pt_1.5_Ni_1−x_/Ni–N–C** | **0.05** | **150 Kpa** | **910** | **N.A.** | **2^[21]^** |
| **Pt-Ni-Au NWs** | **0.10** | **0 Kpa** | **714** | **27.3 (after 10k)** | **3^[22]^** |
| **PtCo/KB-NH_2_** | **0.10** | **150 Kpa** | **960** | **26.7 (after 30k)** | **4^[23]^** |
| **Pt3Co/FeN4-C** | **0.10** | **150 Kpa** | **950** | **10.5 (after 30k)** | **5^[24]^** |
| **L1_0_-PtZn/Pt-C** | **0.104** | **150 Kpa** | **800** | **7 (after 30k)** | **6^[25]^** |
| **Pt1Co1-IMC@Pt/C** | **0.10** | **200 Kpa** | **1060** | **15.1 (after 30k)** | **7^[9]^** |
| **L1_2_-Pt_3_Co/NC-Cys** | **0.10** | **150 Kpa** | **950** | **N.A.** | **8^[26]^** |
| **Pt_1_Co_1_-HC@Pt/C** | **0.10** | **150 Kpa** | **800** | **18.7 (after 30k)** | **9^[27]^** |
| **Pt_3_Co@Mn_SA_-NC** | **0.10** | **150 Kpa** | **1200** | **16.7 (after 30k)** | **10^[28]^** |
| **PtCo-IMC/C** | **0.10** | **150 Kpa** | **1150** | **9 (after 30k)** | **11^[29]^** |
| **Pt_1_Ni_1-x_@Ni_x__NC** | **0.10** | **150 Kpa** | **975** | **8.1 (after 30k)** | **This work** |
| **Pt@NC** | **0.10** | **150 Kpa** | **665** | **26.9 (after 30k)** | **This work** |
| **Pt_1_Ni_1_@ NC** | **0.10** | **150 Kpa** | **886.5** | **16.4 (after 30k)** | **This work** |
| **Sigma 20wt% Pt/C** | **0.10** | **150 Kpa** | **830.9** | **22.7 (after 30k)** | **This work** |

**Reference**

[1] P. Giannozzi, O. Andreussi, T. Brumme, O. Bunau, M. Buongiorno Nardelli, M. Calandra, R. Car, C. Cavazzoni, D. Ceresoli, M. Cococcioni, N. Colonna, I. Carnimeo, A. Dal Corso, S. de Gironcoli, P. Delugas, R. A. DiStasio, Jr., A. Ferretti, A. Floris, G. Fratesi, G. Fugallo, R. Gebauer, U. Gerstmann, F. Giustino, T. Gorni, J. Jia, M. Kawamura, H. Y. Ko, A. Kokalj, E. Kucukbenli, M. Lazzeri, M. Marsili, N. Marzari, F. Mauri, N. L. Nguyen, H. V. Nguyen, A. Otero-de-la-Roza, L. Paulatto, S. Ponce, D. Rocca, R. Sabatini, B. Santra, M. Schlipf, A. P. Seitsonen, A. Smogunov, I. Timrov, T. Thonhauser, P. Umari, N. Vast, X. Wu, S. Baroni, *J. Phys.:Condens. Matter* **2017**, 29, 465901.

[2] M. J. van Setten, M. Giantomassi, E. Bousquet, M. J. Verstraete, D. R. Hamann, X. Gonze, G. M. Rignanese, *Comput. Phys. Commun.* **2018**, 226, 39.

[3] I. C. Man, H. Y. Su, F. Calle‐Vallejo, H. A. Hansen, J. I. Martínez, N. G. Inoglu, J. Kitchin, T. F. Jaramillo, J. K. Nørskov, J. Rossmeisl, *ChemCatChem* **2011**, 3, 1159.

[4] J. R. J. K. Nørskov, A. Logadottir, L. Lindqvist, J. R. Kitchin, T. Bligaard, H. Jónsson, *J. Phys. Chem. B*, 108, 17886.

[5] X. Liu, Y. Wang, J. Liang, S. Li, S. Zhang, D. Su, Z. Cai, Y. Huang, L. Elbaz, Q. Li, *J. Am. Chem. Soc.* **2024**, 146, 2033.

[6] S. Shin, E. Lee, J. Nam, J. Kwon, Y. Choi, B. J. Kim, H. C. Ham, H. Lee, *Adv. Energy Mater.* **2024**, 14, 2400599.

[7] Z. Huang, Q. Xiao, T. Ding, J. Xia, C. Zhan, X. Meng, C.-W. Pao, Z. Hu, W.-H. Huang, Y. Wang, N. Chen, L. Cao, X. Huang, *Sci. Adv.* **2025**, 11, eadt4914.

[8] Z. Wang, W. Wu, H. Jiang, S. Chen, R. Chen, Y. Zhu, Y. Xiao, H. Lv, J. Zhong, N. Cheng, *Adv. Funct. Mater.* **2024**, 34, 2406347.

[9] Q. Cheng, S. Yang, C. Fu, L. Zou, Z. Zou, Z. Jiang, J. Zhang, H. Yang, *Energy Environ. Sci.* **2022**, 15, 278.

[10] S.-N. Hu, W.-C. Xu, N. Tian, S.-M. Chen, M.-Y. Li, J.-F. Shen, J.-X. Lin, S.-L. Guo, X.-Y. Huang, Z.-Y. Zhou, S.-G. Sun, *Energy Environ. Sci.* **2024**, 17, 3099.

[11] Y. Pan, Y. Chen, Y. Li, M. Liu, L. Yao, H. Zhao, X. Zhao, Z. Yue, S. Gui, Y. Chen, Q. Cheng, H. Yang, *Adv. Funct. Mater.* **2025**, 35, 2503628.

[12] X. Cao, H. Guo, Y. Han, M. Li, C. Shang, R. Zhao, Q. Huang, M. Li, Q. Zhang, F. Lv, H. Tan, Z. Qian, M. Luo, S. Guo, *Nat. Commun.* **2025**, 16, 2851.

[13] D. Xue, Y. Yuan, Y. Yu, S. Xu, Y. Wei, J. Zhang, H. Guo, M. Shao, J. N. Zhang, *Nat. Commun.* **2024**, 15, 5990.

[14] L. Tian, X. Gao, M. Zhu, Z. Huang, B. Wu, C. Chen, X. Ma, Y. Ruan, W. Guo, X. Meng, H. Wang, W. Du, S. He, H. Pan, X. Zheng, Z. Wu, H. Zhou, J. Xia, Y. Wu, *Adv. Mater.* **2025**, 37, e2417095.

[15] R. Gui, H. Cheng, M. Wang, X. Tai, H. Zhang, C. Liu, X. Cao, C. Chen, M. Ge, H. Wang, X. Zheng, W. Chu, Y. Lin, Y. Xie, C. Wu, *Adv. Mater.* **2024**, 36, e2307661.

[16] Y. Hu, M. Lu, G. Zhang, X. Zhao, Y. Liu, X. Yang, X. Yu, X. Zhang, Z. Lu, L. Li, *Appl. Catal., B* **2025**, 360, 124556.

[17] Y. Jiang, Q. Zhang, J. Qian, Y. Wang, Y. Mu, Z. Zhang, Z. Li, T. Zhao, B. Liu, L. Zeng, *ACS Catal.* **2024**, 14, 6992.

[18] J. Luo, S. Zhang, F. Liu, H. Cui, X. Liu, H. Liao, Y. Gu, M. Liu, P. Tan, J. Pan, *Adv. Funct. Mater.* **2025**, 35, 2422533.

[19] S. Zhou, W. Bi, J. Zhang, L. He, Y. Yu, M. Wang, X. Yu, Y. Xie, C. Wu, *Adv. Mater.* **2024**, 36, e2400808.

[20] J. Cao, H. Cao, F. Wang, H. Zhu, *J. Power Sources* **2021**, 489, 229425.

[21] W. Guo, X. Gao, M. Zhu, C. Xu, X. Zhu, X. Zhao, R. Sun, Z. Xue, J. Song, L. Tian, J. Xu, W. Chen, Y. Lin, Y. Li, H. Zhou, Y. Wu, *Energy Environ. Sci.* **2023**, 16, 148.

[22] Z. Lin, Y. Sheng, J. Li, Z. Rui, Y. Liu, J. Liu, Z. Zou, *Chem. Commun.* **2020**, 56, 4276.

[23] Q. Gong, H. Zhang, H. Yu, S. Jeon, Y. Ren, Z. Yang, C.-J. Sun, E. A. Stach, A. C. Foucher, Y. Yu, M. Smart, G. M. Filippelli, D. A. Cullen, P. Liu, J. Xie, *Matter* **2023**, 6, 963.

[24] Z. Qiao, C. Wang, C. Li, Y. Zeng, S. Hwang, B. Li, S. Karakalos, J. Park, A. J. Kropf, E. C. Wegener, Q. Gong, H. Xu, G. Wang, D. J. Myers, J. Xie, J. S. Spendelow, G. Wu, *Energy Environ. Sci.* **2021**, 14, 4948.

[25] J. Liang, Z. Zhao, N. Li, X. Wang, S. Li, X. Liu, T. Wang, G. Lu, D. Wang, B. J. Hwang, Y. Huang, D. Su, Q. Li, *Adv. Energy Mater.* **2020**, 10, 2000179.

[26] J. Liang, H. Yu, M. J. Zachman, S. Hwang, M. Qi, Y. Zeng, B. Zhang, J. Li, J. Guo, C. Dun, N. Macauley, G. Wu, *Adv. Mater.* **2025**, 10.1002/adma.202510847, e10847.

[27] M. I. Maulana, H.-Y. Lee, C. Gyan-Barimah, J. H. Sung, J.-S. Yu, *J. Mater. Chem. A* **2024**, 12, 27979.

[28] Y. Zeng, J. Liang, C. Li, Z. Qiao, B. Li, S. Hwang, N. N. Kariuki, C. W. Chang, M. Wang, M. Lyons, S. Lee, Z. Feng, G. Wang, J. Xie, D. A. Cullen, D. J. Myers, G. Wu, *J. Am. Chem. Soc.* **2023**, 145, 17643.

[29] Q. Cheng, T. Wang, Y. Chen, Y. Pan, Y. Chen, B. Yang, H. Yang, *eScience* **2025**, 6, 100453.
